# Subgroup level differences of physiological activities in marine Lokiarchaeota

**DOI:** 10.1038/s41396-020-00818-5

**Published:** 2020-11-04

**Authors:** Xiuran Yin, Mingwei Cai, Yang Liu, Guowei Zhou, Tim Richter-Heitmann, David A. Aromokeye, Ajinkya C. Kulkarni, Rolf Nimzyk, Henrik Cullhed, Zhichao Zhou, Jie Pan, Yuchun Yang, Ji-Dong Gu, Marcus Elvert, Meng Li, Michael W. Friedrich

**Affiliations:** 1 Faculty of Biology/Chemistry, University of Bremen, Bremen, Germany; 2 MARUM, Center for Marine Environmental Sciences, University of Bremen, Bremen, Germany; 3 Shenzhen Key Laboratory of Marine Microbiome Engineering, Institute for Advanced Study, Shenzhen University, Shenzhen, China; 4 Key Laboratory of Optoelectronic Devices and Systems of Ministry of Education and Guangdong Province, College of Optoelectronic Engineering, Shenzhen University, Shenzhen, China; 5 International Max-Planck Research School for Marine Microbiology, Max Planck Institute for Marine Microbiology, Bremen, Germany; 6 Laboratory of Environmental Microbiology and Toxicology, School of Biological Sciences, The University of Hong Kong, Pokfulam Road, Hong Kong SAR, China

**Keywords:** Archaeal physiology, Stable isotope analysis, Microbial ecology

## Abstract

Asgard is a recently discovered archaeal superphylum, closely linked to the emergence of eukaryotes. Among Asgard archaea, Lokiarchaeota are abundant in marine sediments, but their in situ activities are largely unknown except for *Candidatus* ‘Prometheoarchaeum syntrophicum’. Here, we tracked the activity of Lokiarchaeota in incubations with Helgoland mud area sediments (North Sea) by stable isotope probing (SIP) with organic polymers, ^13^C-labelled inorganic carbon, fermentation intermediates and proteins. Within the active archaea, we detected members of the Lokiarchaeota class Loki-3, which appeared to mixotrophically participate in the degradation of lignin and humic acids while assimilating CO_2_, or heterotrophically used lactate. In contrast, members of the Lokiarchaeota class Loki-2 utilized protein and inorganic carbon, and degraded bacterial biomass formed in incubations. Metagenomic analysis revealed pathways for lactate degradation, and involvement in aromatic compound degradation in Loki-3, while the less globally distributed Loki-2 instead rely on protein degradation. We conclude that Lokiarchaeotal subgroups vary in their metabolic capabilities despite overlaps in their genomic equipment, and suggest that these subgroups occupy different ecologic niches in marine sediments.

## Introduction

Lokiarchaeota, previously described as Marine Benthic Group B or Deep-Sea Archaeal Group [1–3], belong to the recently proposed archaeal superphylum Asgard, together with the phyla Thorarchaeota [4], Odinarchaeota, Heimdallarchaeota, Helarchaeota [5], and Gerdarchaeota [6]. The discovery of genes of eukaryotic signature proteins in Asgard opened new perspectives on the evolution of eukarya [5, 7]. Specifically, Lokiarchaeota appear to be widely dispersed and highly abundant and diverse across many coastal and deep-sea marine sediments [2, 8], indicating a high ecological plasticity.

The presence of a tetrahydromethanopterin-dependent Wood-Ljungdahl (H_4_MPT-WL) pathway for inorganic carbon utilization is a known feature of Lokiarchaeota [9]. Organic carbon may be utilized as well in various ways as the presence of genes encoding for the β-oxidation of fatty acids and various pathways for carbohydrates, peptide and amino acid degradation suggests [10]. Besides, recent studies have discovered that some Lokiarchaeota are homoacetogens [9] and one Lokiarchaeum has been described to syntrophically grow with methanogens or sulfate-reducing bacteria while degrading amino acids [11]. These findings hint at substantial variation within the physiology of Lokiarchaeota. Still, there is a dearth of evidence on the in situ lifestyles and ecological roles of Lokiarchaeota, and it is unclear whether subgroups classified in this phylum differ in their physiological properties [8], which complicates the specific assignment of carbon and energy metabolisms to Lokiarchaeota.

The detection of functional genes in metagenome-assembled genomes (MAGs) of as yet mostly uncultivated archaea suggests metabolic potentials that still await verification in many cases. Probing potential physiologies of uncultivated archaea under close to in situ conditions is feasible by stable isotope probing (SIP), e.g. of nucleic acids [12]. Using RNA-SIP facilitates ultra-sensitive labelling with a detection threshold below 0.001% for fully ^13^C-labelled nucleic acids [13, 14]. Thus, in order to illuminate the in situ metabolic capabilities and activities of uncultivated archaea including Lokiarchaeota in marine sediments, we used both RNA-SIP and DNA-SIP techniques in combination with various ^13^C-labelled substrates. We hypothesized that (1) Lokiarchaeota can utilize an array of carbon sources, which are widely available in marine sediments. (2) However, not all clades within Lokiarchaeota have the same metabolic capabilities. In combination with metagenomic analysis, we found that distinct Lokiarchaeota subgroups are specialized in the degradation of different classes of organic compounds.

## Methods

### Sediment incubation setup for SIP

Sediment for incubations was collected from Helgoland mud area (54°05.23′N, 007°58.04′E) by gravity coring during the RV HEINCKE cruise HE443 in 2015. Sediment cores were kept at 4 °C on board during the cruise; in the home laboratory, cores were sectioned into 25-cm depth intervals, and sediment was stored at 4 °C in 2.6-L-glass jars, overlain with anoxic artificial seawater and headspace flushed with N_2_. Geochemical profiles were described previously [15]. Sediment from the methanic zone (238–263 cm depth) and sulfate free artificial seawater (w:v = 1:4, 50 ml) were homogenized and incubated anaerobically in sterile 120-ml serum bottles sealed with butyl rubber stoppers, and headspace flushed with N_2_. A 10-day pre-incubation was performed by exchanging headspace with N_2_ to remove CO_2_. Three different incubation setups were used to address our hypotheses regarding carbon utilization modes: (1) Addition of ^13^C-DIC and organic carbon or sulfur to account for the capability of Lokiarchaeota to assimilate inorganic carbon; (2) Addition of ^13^C-fermentation products (without ^13^C-DIC) to detect utilization of fermentation products; (3) Addition of a combination of protein (^13^C-labelled and unlabelled) and DIC (^13^C-labelled and unlabelled) to check for mixotrophic use of both, protein and DIC as carbon sources. For incubations fed with organic polymers and sulfur, triplicate setups were supplemented with unlabelled electron donors (1 g/l sulfur, 30 mg/l lignin, 30 mg/l humic acid, and 30 mg/l cellulose separately), electron acceptors (30 mM lepidocrocite) and 10 mM sodium bicarbonate (99% ^13^C-labelled bicarbonate provided by Cambridge Isotope Laboratories, Tewksbury, Massachusetts, USA). Sediment slurries were incubated at 10 °C. An additional setup fed with unlabeled bicarbonate was used as a control for comparison. Because of the very slow growth rate of Lokiarchaeota [11], samples collected on day 255 were used for RNA-SIP analysis and those on day 386 were used for DNA-SIP analysis based on the development of the stable carbon isotopic composition of total organic carbon (δ^13^C-TOC) as a proxy for carbon assimilation activity in incubations. The δ^13^C values of TOC were measured on a Flash 2000 elemental analyzer coupled with DELTA V Plus IRMS via a ConFlow II interface (EA-IRMS, Thermo Scientific, Bremen, Germany). Prior to analysis, dried sediment from 0.5 ml slurry was acidified using 1 ml HCl (37%) overnight to remove inorganic carbon and followed by evaporation for several days until HCl acid was fully evaporated.

Separate RNA-SIP incubations amended with ^13^C-labelled fermentation intermediates (acetate, propionate, lactate and butyrate; 99%, all C-atoms ^13^C-labelled, sodium salts) were setup in triplicates using Helgoland mud area sediment from methanic zone (95–120 cm). The corresponding unlabelled fermentation intermediates were used as control incubations. Low concentrations of fermentation intermediates (~60 µM carbon) were used, i.e. 30 µM acetate, 20 µM propionate, 20 µM lactate and 15 µM butyrate. δ^13^C-CO_2_ in headspace of SIP incubations was monitored as previously published (Fig. S1) [16]. Incubations were stopped after up to 13 days according to the increase of δ^13^C-CO_2_ in headspace (Fig. S1a) and the low starting concentration of amended fermentation intermediates.

Protein utilization by Lokiarchaeota was tested using RNA-SIP incubations with upper layer sediment (16–36 cm) and ^13^C-labelled protein. Labelled ^13^C-protein was obtained by growing *Escherichia* *coli* DSM 498 strain in both unlabelled and ^13^C-labelled *E. coli*-OD2 C medium (^13^C, 98%, Silantes, Germany) [17]. Protein extraction was performed as previously described with modifications [18]. Briefly, after harvesting, *E. coli* cells were disintegrated by bead beating in phosphate buffer and Tris-NaCl-sodium dodecyl sulfate solution, and nucleic acids in the upper aqueous phase were removed after treatment with phenol:chloroform:isoamylalcohol (25:24:1; v/v/v). The protein layer at the aqueous-organic interphase was collected, washed with 1 ml DEPC-treated water to remove residue nucleic acids, followed by chloroform:isoamylalcohol (24:1; v/v) washing to remove phenol and lipids from the protein layer. The protein pellet was dried under the fume hood and mixed with autoclaved deionized water. Dissolved protein was quantified using the protein assay kit (Invitrogen, Eugene, Oregon, USA). DNA contamination was checked with the Quant-iT PicoGreen assay (Invitrogen, Eugene, Oregon, USA) (<0.6 ng/µl in all cases). Triplicate incubations were conducted by amending ~100 µg dissolved ^13^C-labelled protein. Streptomycin (100 mg/l) was used in incubations to inhibit bacterial activity. To estimate inorganic carbon utilization by Lokiarchaeota, 10 mM ^13^C-labelled bicarbonate (^13^C-DIC) was supplemented to incubations after 11 days (Fig. S1b) in order to minimize ^13^C-DIC based cross-feeding. Incubations amended with unlabelled protein and DIC were used as control. All incubations were stopped after 24 days based on the measurement of δ^13^C-CO_2_ in the headspace [16].

For all SIP incubation samples, DNA and RNA extraction were performed in triplicate as described in detail in the Supplementary Methods.

### Isopycnic centrifugation, gradient fractionation and 16S rRNA gene sequencing

Isopycnic centrifugation and gradient fractionation were performed to separate ^13^C-labelled from unlabelled nucleic acids as previously described [19]. About 0.3–0.7 µg RNA and 4–6.5 µg DNA were used for RNA- and DNA-SIP, respectively. Isopycnic centrifugation and gradient fractionation are described in Supplementary Methods. After ultracentrifugation, 13 fractions (~400 µl) were obtained from each sample. RNA was reversely transcribed using the high capacity cDNA reverse transcription kit (Applied Biosystems, Foster City, California, USA). cDNA from fractions 4 and 5 (heavy), 6 and 7 (middle), 8 and 9 (light), as well as 10 and 11 (ultra-light) were combined for sequencing. DNA samples from several fractions without pooling were used for high-throughput sequencing. PCR targeting the V4 region of 16S rRNA gene sequences was performed with KAPA HiFi HotStart PCR kit (KAPA Biosystems, Cape Town, South Africa) and barcoded archaeal primer Arc519F (5′-CAGCMGCCGCGGTAA-3′) [20] and Arch806R (5′-GGACTACVSGGGTATCTAAT-3′) [21]. Thermocycling was as follows: 95 °C for 3 min; 35 cycles at 98 °C for 20 s, 61 °C for 15 s, and 72 °C for 15 s; 72 °C for 1 min. PCR products were purified using the Monarch PCR Cleanup Kit (New England Biolabs, Ipswich, Massachusetts, USA) according to the manufacturer. Equimolar amounts of amplicons per sample were combined based on PicoGreen quantification. For SIP samples from incubations amended with DIC, amplicons were sequenced using Illumina Hiseq 4000 platform with 150-bp paired-end reads at GATC Biotech (Konstanz, Germany). cDNA of RNA-SIP samples of incubations amended with fermentation intermediates and protein were sequenced using Novaseq6000 platform with 250-bp paired-end reads at Novogene (Cambridge, UK). Raw reads were processed using the QIIME 1.9.0 software package [22]. OTUs were clustered at 97% identity using UPARSE-OTU [23]. Sequencing data of SIP samples have been submitted to Short Reads Archive with accession numbers from SRR8607872 to SRR8607991 and SRR11429462 to SRR11429436.

### Criteria for identifying SIP fractions containing ^13^C-labelled nucleic acids

Label incorporation into distinct OTUs was detected by the presence of nucleic acid templates in heavy gradient fractions. For OTUs with a high background in ^12^C-DIC incubations (Loki-3), we have used three criteria to define isotopic enrichment of nucleic acids following recommendations by Lueders [24]: (1) A more than 5% increase of relative abundance of OTUs in the heavy fractions of amended incubations compared to the maximum relative abundance of the control incubations (inter-gradient comparison); (2) A more than 5% increase of relative abundance of OTUs between light and heavy fractions (intra-gradient evaluation); (3) Defining SIP fractions containing ^13^C-labelled nucleic acids by standardization with RNA and DNA of fully labelled and unlabeled *E. coli* standards (Fig. S2). Based on these standard gradients, densities starting from 1.797 g/ml (RNA-SIP) and from 1.702 g/ml (DNA-SIP) mark the incorporation of ^13^C-labelled nucleic acids when first two criteria were met, with increasing densities representing higher degrees of labelling efficiency (see Supplementary Methods). Since the G+C mol% content of Loki-2 and Loki-3 DNA was ~30% (based on MAGs, Table S1), partitioning of unlabeled DNA from these target archaea into fractions with higher density was ruled out. For RNA, density effects were unlikely due to a narrow range of G+C mol% content in rRNA of 50–60% [24].

### Phylogenetic analysis of Lokiarchaeotal 16S rRNA genes

Archaeal 16S rRNA gene sequences were aligned using SINA Aligner [25]. These archaeal sequences included 16S rRNA gene OTUs from Illumina sequencing of RNA-SIP samples, clone sequences from the heavy fraction of DNA-SIP samples, 16S rRNA gene extracted from the six Lokiarchaeotal MAGs and Lokiarchaeota representative sequences obtained from previously studies [8, 11, 26]. Maximum-likelihood tree was inferred with RAxML (version 8.2.11) using the GTRGAMMA model with 1000 times rapid bootstrapping [27]. The tree files were visualized using iTOL software [28] and edited in Adobe Illustrator. Calculation of identity of 16S rRNA gene clones was performed in ARB [29]. Fragments of 16S rRNA gene from position of *E. coli* 29 to 796 (~770 bp) were used for calculating the identity (Table S2).

### Metagenomic and metatranscriptomic analysis

DNA from the heavy fraction with a density of 1.719 g/ml (the second heaviest fraction, from which template could be amplified) from the incubations amended with sulfur/lepidocrocite and original DNA extracts from Helgoland sediment at depth from 238 to 263 cm were used for metagenomic sequencing at Novogene (Cambridge, UK) using Illumina HiSeq sequencing with 150-bp paired-end reads. Coastal sediment samples from South China were used for both metagenomic and metatranscriptomic analysis (see Supplementary Methods for more details). Raw metagenomic DNA reads were de-replicated and trimmed using the script “dereplicate.pl” and sickle (version 1.33) [30] with the option “-q 25”, respectively. Paired-end Illumina reads for each sample were de novo assembled using IDBA-UD (version 1.1.1) [31] with the parameters “-mink 65, -maxk 145, -steps 10”. Scaffolds were binned into genomic bins with trimmed reads using a combination of MetaBAT2 [32] and Das Tool [33]. Briefly, 12 sets of parameters were set for MetaBAT2 binning [34], and Das Tool was further applied to obtain an optimized, non-redundant set of bins. To improve the quality of the bins (e.g. scaffold length and bin completeness), each Lokiarchaeotal MAG was remapped with the short-read mapper BWA [35] and re-assembled using SPAdes (version 3.0.0) [36] or IDBA-UD (version 1.1.1) [31], followed by MetaBAT2 and Das Tool binning. Lokiarchaeotal MAGs with high contamination were further refined with Anvi’o software (version 2.2.2) [37]. The completeness, contamination and strain heterogeneity of the genomic bins were estimated by CheckM (version 1.0.7) software [38]. Lokiarchaeota MAGs were described in Table S1.

Protein-coding regions were predicted using Prodigal (version 2.6.3) with the “-p meta” option [39]. The KEGG server (BlastKOALA) [40], eggNOG-mapper [41], InterProScan tool (V60) [42] and BLASTp vs. NCBI-nr database searched on December 2017 (*E*-value cutoff ≤ 1e−5) were used to annotate the protein-coding regions (Table S3). The Lokiarchaeotal MAGs and metatranscriptomic data are available in NCBI database under the project PRJNA495098, PRJNA360036 and PRJNA505997.

### Phylogenetic analyses of Lokiarchaeotal MAGs

The 16S rRNA gene sequences and a concatenated set of 122 archaeal-specific conserved marker genes [43, 44] were used for phylogenetic analyses of Lokiarchaeota. Ribosomal RNA genes in the MAGs were extracted by Barrnap (version 0.3, http://www.vicbioinformatics.com/software.barrnap.shtml). Marker genes for phylogenomic tree were identified using hidden Markov models and were aligned separately using hmmalign from HMMER3 [45] with default parameters. The 122 archaeal marker genes were identified using hidden Markov models. Each protein was individually aligned using hmmalign [46]. The concatenated alignment was trimmed by BMGE with flags “-t AA -m BLOSUM30” [47]. Then, maximum-likelihood trees were built using IQ-TREE with the best-fit model of “LG + I + G4” followed by extended model selection with FreeRate heterogeneity and 1000 times ultrafast bootstrapping.

## Results

### Identification and carbon utilization of metabolically active Lokiarchaeota

We studied the carbon metabolism of active Lokiarchaeota in incubations with Helgoland mud sediment, in which up to 10% of archaeal sequences were previously identified as Lokiarchaeota [48]. We applied both, RNA and DNA based SIP by using ^13^C-labelled bicarbonate (DIC) in combination with different electron donors (sulfur, lignin, humic acids and cellulose) and/or the iron oxide lepidocrocite (γ-FeO(OH)) as electron acceptor, all of which are detectable in marine sediments [15, 49].

**Fig. 1:**
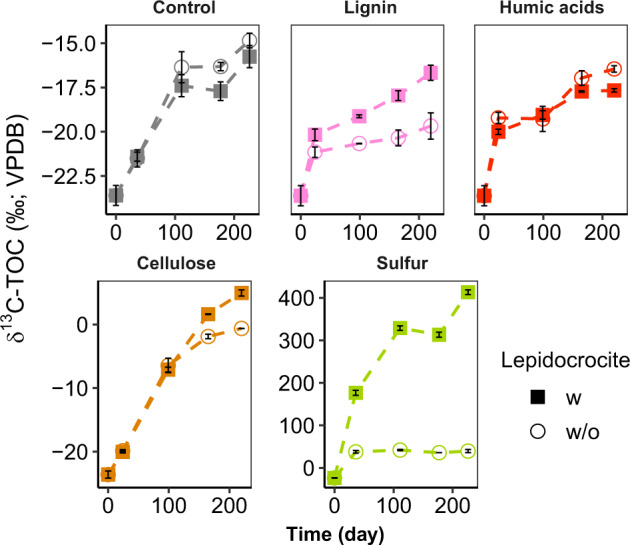
Development of δ^13^C values of TOC in SIP incubations. All incubations were amended with ^13^C-DIC (*n* = 3, error bar = SD).

Inorganic carbon assimilation is an ideal proxy for monitoring microbial activity since both autotrophs and heterotrophs incorporate CO_2_ into biomass [50, 51]. Microbial activity in SIP incubations amended with ^13^C-DIC was followed by determining the δ^13^C-TOC (Fig. 1). In contrast to control incubations with only ^13^C-DIC, the addition of unlabeled cellulose and sulfur substantially increased δ^13^C values of TOC above natural abundance to ~30‰ and 428‰, respectively. For aromatic compounds (lignin and humic acids), δ^13^C of TOC was close to control incubations (−20 to −15‰). The amendment of lepidocrocite led to an increase of δ^13^C values of TOC in combination with cellulose and especially sulfur (∆δ^13^C 428‰).

**Fig. 2:**
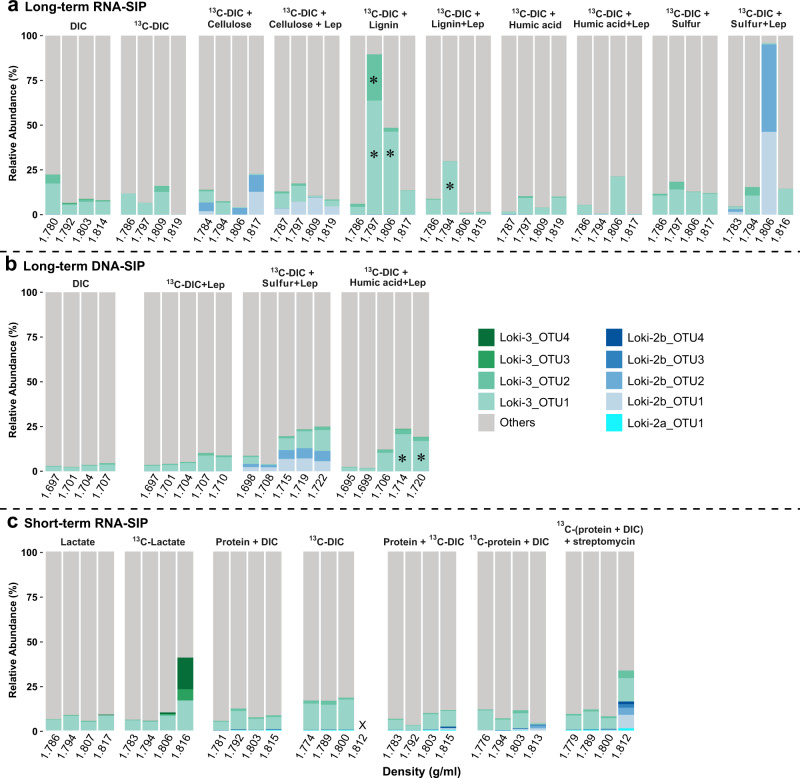
Total sum scaling charts of Lokiarchaeota abundances of archaeal 16S rRNA gene sequences from selected “light” and “heavy” gradient fractions. **a** Long-term RNA-SIP samples amended with ^13^C-DIC, **b** Long-term DNA-SIP incubations amended with ^13^C-DIC, **c** Short-term RNA-SIP samples from lactate and protein incubations. Differences in *x*-axis scales between RNA and DNA-SIP are due to the different gradient media used (CsTFA vs CsCl, respectively). For RNA-SIP, pairs of fractions (fractions 4 and 5, 6 and 7, 8 and 9, 10 and 11) were combined for Illumina sequencing, whereas individual fractions were used for DNA-SIP. Density was indicated as the average density of combined fractions for RNA-SIP samples. Due to density differences between RNA and DNA, the threshold density fractions to delineate ^13^C-labelled nucleic acids differ between RNA (>1.797 g/ml) and DNA (>1.702 g/ml). “X” indicates that cDNA synthesis failed because of insufficient amount of RNA in these fractions. For the Loki-2 OTUs which were not detectable in controls, label incorporation activity was detected by their presence in heavy fractions. An asterisk indicates inter-gradient increase of Loki-3 OTUs (see Fig. S3 for intra-gradient assessment; both approaches were in agreement). DNA with densities >1.71 g/ml was not obtained from DIC incubations. Lep lepidocrocite. DIC dissolved inorganic carbon, i.e. bicarbonate.

Using nucleic acid-SIP, we identified nine active Lokiarchaeotal OTUs (97% 16S rRNA sequence identity cutoff) in incubations fed with various electron donors and ^13^C-DIC (Fig. 2). Prominently, OTUs previously classified as Loki-2b [8] were found in high abundances in the RNA-SIP fractions containing ^13^C-RNA for setups with unlabeled cellulose, with ~20 and 10% of total archaeal sequences at density of 1.817 and 1.809 g/ml for treatment of cellulose and cellulose/lepidocrocite, respectively. Loki-2b were also identified in incubations amended with sulfur/lepidocrocite (~90% at 1.806 g/ml) (Fig. 2a), for which we saw also incorporation into isotopically “heavy” DNA (Fig. 2b). However, Loki-2b remained undetected in both, incubations of unlabelled DIC control and ^13^C-DIC control. In comparison to the control incubations, Loki-3 were stimulated in incubations amended with lignin, but here, the RNA was isotopically separated into partially labelled fractions (~50–90% of all archaeal sequences in fractions with density of 1.797 and 1.806 g/ml) (Figs. 2a and S3). In addition, DNA of Loki-3 OTUs was recovered from ^13^C-labelled fractions (>1.706 g/ml) of ^13^C-DIC/humic acid/lepidocrocite incubations after comparison with both unlabelled DIC and ^13^C-DIC/lepidocrocite (Figs. 2b and S3).

Since Lokiarchaeota were stimulated by organic polymers, we checked whether Lokiarchaeota used intermediates of polymer fermentation formed by other microorganisms. In RNA-SIP incubations with ^13^C-labelled short-chain fatty acids (acetate, propionate and butyrate), we detected Loki-3, but no Loki-2b OTUs in both heavy and light fractions (Figs. 2c and S2e). In incubations with ^13^C-lactate, previously undetected Loki-3 OTUs i.e. OTUs 3 and 4, were found in heavy gradient fractions (>1.816 g/ml) after 8 days of incubations (Figs. 2c and S1a).

Loki-2b archaea were identified only in incubations with high microbial activity and high δ^13^C values of TOC, which suggests this group utilizes biomass compounds such as proteins or amino acids as carbon sources and thus cross-feeding has occurred representing potentially a web food interaction in these incubations [11]. Hence, RNA-SIP incubations supplemented with protein were conducted to further prove the dependence of Loki-2b on microbial biomass. In incubations with ^13^C-protein, Loki-2b OTUs were enriched in heavy fractions (Fig. 2c and Table S4). We also found Loki-2b OTUs being labelled when ^13^C-DIC and unlabelled protein were amended (Fig. 2c and Table S4). Furthermore, a new subgroup of Loki-2, i.e. Loki-2a, was detected at low abundance in heavy fractions of incubations with ^13^C-protein, ^13^C-DIC and streptomycin (Fig. 2c).

**Fig. 3:**
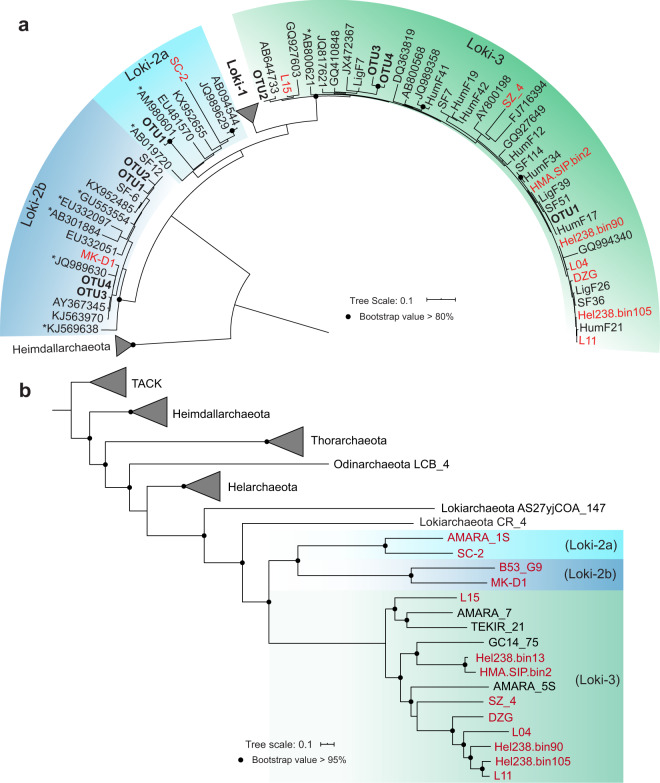
Maximum-likelihood phylogeny of Lokiarchaeota. **a** Maximum-likelihood tree of Lokiarchaeotal 16S rRNA genes. 16S rRNA sequences and OTUs obtained in the present study were marked in bold, and those extracted from MAGs were marked in red. SF: clones from incubation amended with sulfur and lepidocrocite; HumF: clones from incubation amended with humic acid and lepidocrocite; LigF: clones from incubation amended with lignin and lepidocrocite. Clone library construction is described in the Supplementary Methods. MK-D1: Lokiarchaeota MAG obtained from enrichment [11]. L15, L04, L11: 16S rRNA genes from near-complete Lokiarchaeota MAGs obtained from a previous study [26]. An asterisk indicates the same reference sequences used for 16S rRNA gene tree construction with a previous study using Namibian sediments [9]. **b** Maximum-likelihood tree of Lokiarchaeotal MAGs inferred from a concatenated alignment of 122 archaeal marker genes and re-rooted with superphylum TACK. MAGs Hel238.bin13, Hel238.bin105 and Hel238.bin90 were obtained from Helgoland Mud sediment; MAG HMA.SIP.bin2 was obtained from heavy DNA of SIP incubations amended with sulfur, lepidocrocite and ^13^C-DIC; MAGs SZ_4 and DZG were retrieved from original sediment of South China Sea (Table S1). Information of all Lokiarchaeota MAGs was described in Table S1.

The identification accuracy of Lokiarchaeota subgroups with short Illumina sequences was checked with a maximum-likelihood tree containing long 16S rRNA gene fragments (~770 bp), obtained from metagenomic assembly and a clone library established from the ^13^C-labelled DNA-SIP fractions (Fig. 3a). Indeed, Loki-2b, Loki-2a and Loki-3 were phylogenetically different, whereas Loki-3 was found to be phylogenetically more diverse than the other two subgroups. Clones and MAGs of Loki-2 and Loki-3 16S rRNA genes were on average 83% identical, allowing the assignment of a class level difference between these two subgroups (Table S2) [52].

### Physiological capabilities revealed by metagenome and metatranscriptome analysis

We used isotopically heavy DNA fractions from SIP-incubations together with native DNA from coastal sediments containing Lokiarchaeota for deep metagenomic sequence analysis (Table S1). Seven Lokiarchaeotal MAGs with genome completeness above 70% were recovered (Table S1). These Lokiarchaeotal MAGs were identified as Loki-3 and Loki-2 members according to phylogenetic and phylogenomic analyses (Fig. 3), supported by the high average identity of nucleotides (ANI > 69%) and amino acids (AAI > 62%) within Loki-3 but lower ANI (<65%) and AAI (<48%) between the two subgroups (Loki-3 and 2b) (Fig. S4).

**Fig. 4:**
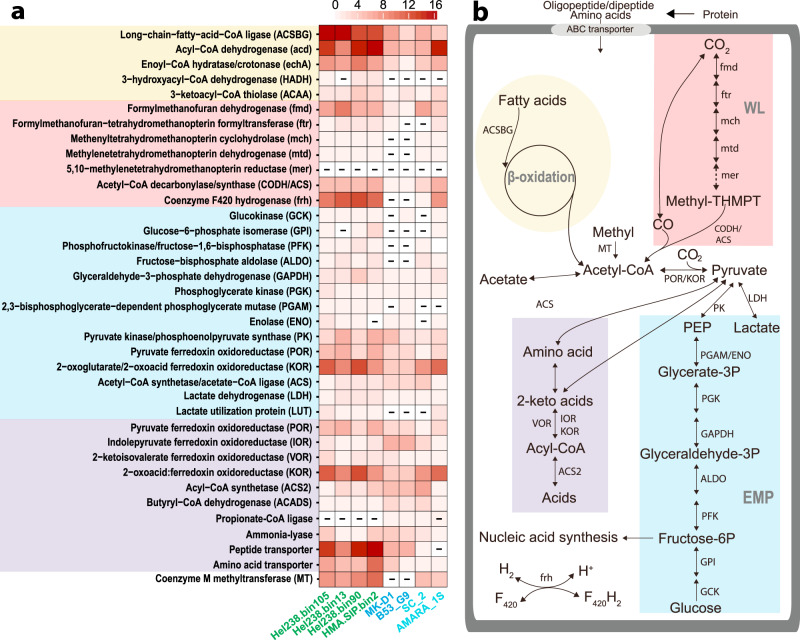
Key genes and metabolic pathways in Lokiarchaeota. **a** Number of gene homologues in Lokiarchaeota MAGs. Lokiarchaeota MAGs marked with green indicate Loki-3 obtained from Helgoland sediment and sediment incubations. MAGs marked with blue and cyan indicate Loki-2b and Loki-2a, respectively (see Table S1 for detail MAG information). MK-D1: *Candidatus* ‘Prometheoarchaeum syntrophicum’ strain MK-D1 [11]. Symbol “-“ indicates absence of gene in MAGs. **b** Proposed active pathway in Lokiarchaeota (Loki-3). Pathways were constructed based on Lokiarchaeotal MAGs obtained from this study (Table S3). EMP Embden–Meyerhof–Parnas pathway, WL tetrahydromethanopterin-dependent Wood-Ljungdahl pathway. Incomplete pathways were indicated by dashed line. Pathway names associated with the colours: yellow: β-oxidation; pink: WL; light blue: EMP; purple: amino acid degradation.

We analyzed nine Loki-3 MAGs and four Loki-2 MAGs, including MAGs from SIP incubations, Helgoland Mud sediments, South China sediments, in combination with MAGs from previous studies [11, 26] (Tables S3, S5). According to metagenomic inference, all Loki-3 MAGs harboured similar pathways and capabilities, including complete pathways for β-oxidation of long-chain fatty acids, amino acid degradation and glycolysis via the Embden–Meyerhof–Parnas (EMP) pathway (Fig. 4 and Table S5). Loki-3 have an almost complete H_4_MPT-WL, as all six MAGs obtained in our study lacked genes encoding the typical 5,10-methylenetetrahydromethanopterin reductase (*mer*); however, a *mer* gene candidate within the same protein family of COG2141 was found (Fig. S5 and Tables S3, S5). Both gene sets for the conversion of lactate to pyruvate, i.e. lactate dehydrogenase and lactate utilization proteins, were identified in Lokiarchaeota MAGs. The 2-keto acid oxidoreductases, which potentially catalyse 2-keto acid activation to acyl-CoA [53], coenzyme M methyltransferase for methyl compounds utilization, as well as multiple coenzyme A ligases including long-chain-fatty-acid-CoA (ACSBG), 4-coumarate-CoA and phenylacetate-CoA ligases were also found (Figs. 4a and S6).

In order to compare the two subgroups of Lokiarchaeota, Loki-3 and -2, we analysed the number of homologues of key genes of β-oxidation, H_4_MPT-WL, EMP and amino acid degradation. Homologues of genes for β-oxidation and EMP pathway and coenzyme M methyltransferase were highly diverse in Loki-3 MAGs compared to Loki-2b (MK-D1) (Fig. 4a and Table S5). In contrast to Loki-3, Loki-2b (MK-D1) had more homologues of genes encoding acyl-CoA synthetase (up to 7) and indolepyruvate ferredoxin oxidoreductase (up to 6) (Fig. 4a and Table S5), which are associated with amino acid degradation. Loki-3 exclusively harbour diverse unique genes involved in sugar and lactate metabolism, methyl utilization as well as β-oxidation (Table S6), while Loki-2,especially for Loki-2b mainly comprise genes related to amino acid degradation such as 2-keto ferredoxin oxidoreductase acyl-CoA synthetase and propionate-CoA ligase (Fig. 4 and Table S5). However, most genes belonging to H_4_MPT-WL and many genes from the EMP pathway were absent in the Loki-2 MAG. Metatranscriptomic analysis showed that the transcripts for most genes involved in the WL pathway and acetyl-CoA carboxylation in Lokiarchaeota were detected in mangrove sediments of South China (Fig. S7).

## Discussion

This study has demonstrated that phylogenetically diverse Lokiarchaeotal subgroups were active in incubations of Helgoland mud sediments when probed with combinations of ^13^C-DIC, organic polymers, sulfur, ^13^C-lactate as well as ^13^C-protein by RNA- and DNA-SIP. This is corroborated by the divergent metagenomic blue prints of the two Lokiarchaeotal subgroups (i.e. Loki-3 and Loki-2) found, which underpin their preferences for distinct carbon sources.

### Identification of active Lokiarchaeota by SIP

Nucleic acid-SIP is a technique used for identifying the active microbial players in incubation studies. However, a valid concern with SIP is the potential for cross-feeding, especially for long-term incubations [54]. To evaluate the possibility of cross-feeding, we followed the change in the stable carbon isotopic composition of TOC in ^13^C-DIC incubations to track biomass formation in incubations since increases in ^13^C-TOC reflect microbial activity [50, 51].

We proved that ^13^C-DIC based cross-feeding i.e. cross-feeding on highly ^13^C-labelled biomass from incubations with ^13^C-DIC, did not occur in incubations amended with ^13^C-DIC/organic polymers for the following reasons: (i) for incubations amended with ^13^C-DIC/lignin and ^13^C-DIC/humic acid/lepidocrocite, δ^13^C of TOC was close to that of control incubations (Fig. 1), indicating a very low activity in these incubations. (ii) Bacteria communities in the heavy fractions specifically in incubations of ^13^C-DIC/lignin and ^13^C-DIC/humic acid/lepidocrocite were not enriched most likely due to the difficulties in aromatic compounds utilization and low growth under anaerobic conditions at low temperature (10 °C) [55, 56] (Fig. S2a). (iii) For incubations amended with ^13^C-DIC/cellulose/lepidocrocite, the organic carbon was unlabelled, rendering heterotrophic cellulose-degrading *Spirochaetaceae* [57] enriched in the light RNA-SIP fractions (Fig. S2), so highly ^13^C-labelled biomass was not produced by bacteria from ^13^C-DIC. Hence, ^13^C-DIC based cross-feeding between bacteria and archaea is unlikely for inorganic carbon assimilation in incubations amended with unlabeled lignin, humic acid and cellulose.

### Flexibility of Loki-3 in carbon utilization

Generally, the evolution of archaea has been hypothesized to be linked to an autotrophic lifestyle [58], and the assimilation of inorganic carbon appears to be important for some archaea because of the pathways involved in biomass formation. Examples include methylotrophic methanogens and Bathyarchaeota when thriving on organic substrates [16, 59]; Bathyarchaeota incorporate substantial amounts of ^13^C-bicarbonate into their tetraether lipids while thriving on lignin as energy source [59]. Likewise, we observed a stimulation of Lokiarchaeota activity and inorganic carbon uptake in the incubations amended with lignin/^13^C-DIC and humic acid/lepidocrocite/^13^C-DIC in RNA- and DNA-SIP incubations (Fig. 2). We ruled out the possibility of Loki-3 utilizing biomass from other cells because no Loki-3 activity was detected when protein was amended. In contrast, Loki-2b, which had an extremely low abundance in DIC controls, was stimulated in the same incubations (Fig. 2c). For those incubations with lignin/^13^C-DIC, high abundances of Loki-3 RNA were found in intermediate, partially labelled RNA-fractions (Fig. S2b). Similarly, Loki-3 were also identified in the fractions containing ^13^C-labelled DNA (Fig. 2b). “Partial labelling” can be the result of mixotrophic metabolism. Certainly, partial labelling can also arise from label dilution and insufficient time for label incorporation. However, the unique activity of Loki-3 in ^13^C-DIC/lignin incubations strongly points to a participation of Loki-3 in lignin degradation. Since the amended organic polymers were unlabelled, the detection of ^13^C-labelled Loki-3 suggests that their biomass was inevitably formed by ^13^C-DIC fixation. Hence, Loki-3 were highly abundant in intermediate, partially labelled fractions of both RNA- and DNA-SIP incubations (e.g. lignin/^13^C-DIC), suggesting mixotrophy by utilizing both, inorganic and organic carbon sources.

**Fig. 5:**
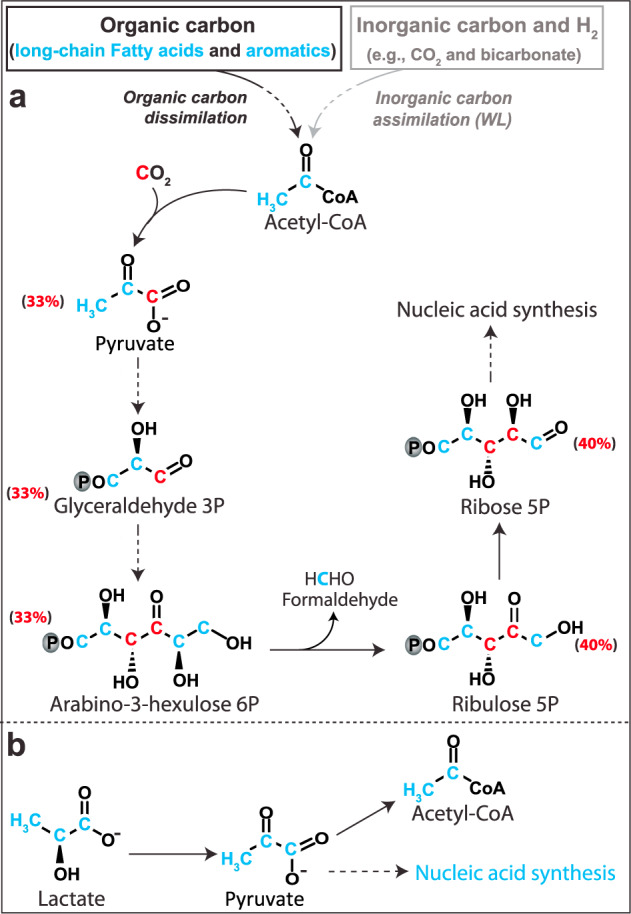
Carbon assimilation patterns into nuleic acid by Lokiarchaeota. **a** Inorganic carbon assimilation into nucleic acids; **b** lactate utilization for nucleic acid synthesis in Loki-3. All genes involved in the biosynthetic pathways of nucleic acids were present in Loki-3 MAGs (Table S3). Labelling levels for each intermediate in (**a**) were based on previous studies [16, 74, 75].

In our incubations, the activity of Loki-3 archaea was triggered by ^13^C-DIC, unlabelled lignin and ^13^C-lactate (indicated by RNA-SIP) as well as by humic acids and lepidocrocite (DNA-SIP), showing a wide range of actively expressed carbon utilization modes. In order to show the effect of labelled carbon on nucleic acids labelling patterns during SIP, nucleic acid synthesis pathways are shown in Fig. 5 according to previous studies [15, 60, 61]. Although the WL pathways found in Lokiarchaeota MAGs might not be complete (or might feature a distant *mer* alternative; Fig. S5), sufficient amounts of inorganic carbon were assimilated into nucleic acids (Fig. 5), suggesting that CO_2_ incorporation is operative in these Lokiarchaeota. When feeding on unlabelled organic carbon such as long-chain fatty acids, aromatic polymers or methyl substrates, generated acetyl-CoA will be used for pyruvate formation after incorporating one CO_2_, elevating the ^13^C-labelling level in pyruvate to 33% (Fig. 5). After cleavage of formaldehyde from arabino-3-hexulose-6-phosphate, this labelling level increases to 40% in ribose during nucleic acid synthesis. This amount of ^13^C in ribose is sufficient for separating labelled from unlabelled RNA [62], consistent with the high abundances of Lokiarchaeota in partially labelled fractions rather than heavy fractions (Fig. 2). Coincidentally, genes involved in WL pathway and acetyl-CoA carboxylation to pyruvate were expressed in the mangrove sediment from South China (Fig. S7). The observation of mixotrophic inorganic carbon assimilation by Lokiarchaeota is consistent with a previous study using Namibian sediments [9]. Hence, CO_2_ incorporation will occur when acetyl-CoA is used for biomass synthesis since formation of pyruvate from acetyl-CoA requires CO_2_ incorporation.

Inorganic carbon assimilation by Loki-3 concomitantly occurred with the input of organic polymers (Fig. 2), indicating the utilization of organic carbon. Considering the low microbial activity indicated by δ^13^C-TOC (Fig. 1) and limited bacterial community shifts in RNA-SIP fractions (Fig. S2a), Loki-3 were likely involved in the degradation of aromatic compounds in SIP incubations. We speculate that organic acids generated from degradation of lignin and humic acids could be used by Loki-3, which is supported by the presence of the complete β-oxidation pathway. The ability to utilize complex organic acids is underpinned by the higher number of homologues of these CoA ligases in Loki-3 than Loki-2b (Figs. 4,  S6 and Table S5). In fact, unlike genes for other pathways, genes encoding all enzymes involved in β-oxidation in Loki-3 were highly expressed, signifying that these pathways are active in situ (Fig. S7 and Table S6). A previous study on the carbon distribution in Helgoland sediments showed that aromatic compound accumulated in deep sediments, suggesting slow degradation of polymeric compounds such as lignin and humics at a depth; moreover, the abundance of Lokiarchaeota increased with depth in these sediments [48]. In addition, the high number of coenzyme M methyltransferase genes in Loki-3 MAGs (Fig. 4a) suggests the possibility of methyl group utilization originating from aromatic polymers.

Fermentation intermediates such as acetate, propionate, butyrate and lactate have been found at concentrations of up to 60 µM in marine sediments [63–65], representing potential carbon sources for Lokiarchaeota. As indicated by RNA-SIP, Loki-3 members were able to use lactate as carbon source at low concentration provided (Fig. 2). For incubations amended with ^13^C-labelled lactate, the strongly labelled RNA of Loki-3 indicated by the specific presence of Loki-3 OTU4 in heavy fractions suggests that lactate was most likely used as sole carbon source while inorganic carbon was not involved in carbon assimilation. Indeed, the nucleic acid synthesis pathway shows that pyruvate formed from lactate under the catalysis of lactate dehydrogenases and lactate utilization proteins can be directly used for ribose formation in the nucleic acid synthesis pathway without inorganic carbon incorporation (Fig. 5). According to phylogenetic analysis, the lactate dehydrogenases of Lokiarchaeota form clusters with homologues of potential lactate utilizers or fermenters in anaerobic sediments such as Clostridia, Atribacteria and *Desulfobacteraceae* (Fig. S8) [66–68]. Genes encoding lactate utilization protein A and B in Lokiarchaeota formed a relatively distant cluster from the other taxonomic groups (Figs. S9 and S10), indicating the specificity of these genes for Lokiarchaeota. The lactate utilization proteins might be expressed preferentially under high availability of iron [69], which is in line with high dissolved iron concentrations (~330 µM) in the Helgoland mud sediment [15]. These lactate utilization proteins were uniquely detected in Loki-3 (Fig. 4 and Table S5), suggesting the specialization of Loki-3 in lactate degradation. The presence of two different lactate utilization systems in Lokiarchaeota underpins that lactate is an important substrate for Lokiarchaeota in Helgoland Mud sediments, in line with a previous study using Namibian sediments [9]. Furthermore, lactate dissimilation to acetate is feasible, since genes encoding acetogenesis from pyruvate were highly expressed (Fig. S7) and lactate in marine sediments can reach concentrations of up to 200 µg/l [64].

We did not observe the utilization of short-chain fatty acids (acetate, propionate and butyrate) and protein by Loki-3 (Fig. S2e). Although we cannot rule out that Loki-3 might use these substrates as carbon and energy source, but because of the short term incubation (8–24 days, Fig. S1) and the generally low activity of Lokiarchaeota, it was not detected by RNA-SIP. Since Loki-3 have been shown to form short-chain fatty acids such as propionate and acetate [9, 11], it is feasible that Loki-3 in Helgoland Mud and mangrove sediment produce volatile fatty acids. At least for lactate fermentation, acetate is a likely product of energy metabolism in Loki-3 (Fig. 4).

### Carbon utilization by Loki-2

**Fig. 6:**
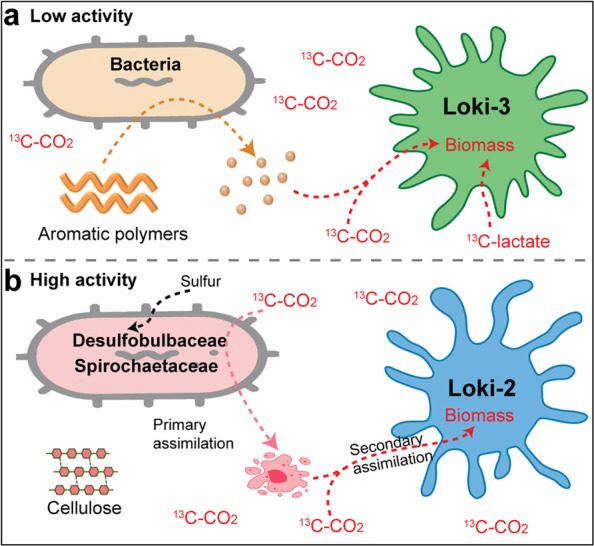
Carbon utilization pattern by Lokiarchaeota in marine sediments. **a** Organic polymer (lignin and humic acids) degradation and potential ecological roles of Loki-3. **b** Carbon utilization of Loki-2 indicated from SIP incubations.

Loki-2b archaea were active specifically in incubations amended with sulfur/lepidocrocite and cellulose when bacterial activity and abundances were high (Fig. 2, Fig. S2). Importantly, Loki-2 including Loki-2a and Loki-2b were stimulated from ^13^C-protein and ^13^C-DIC incubations, providing a link to understand the labelling pattern observed in sulfur/lepidocrocite or cellulose/lepidocrocite incubations. Although amino acid degradation genes were also found in Loki-3 (Fig. 4), RNA-SIP showed that the new Lokiarchaeota subgroup Loki-2 was active rather than Loki-3 when protein was provided (Fig. 2c). Thus, Loki-2 likely used protein, typically representing 50% of the cells dry matter [70], formed by abundantly enriched populations such as members of the families *Desulfobulbaceae* and *Spirochaetaceae*. *Desulfobulbaceae* are known as autotrophic sulfur disproportionating microorganisms [71], and therefore, sulfur disproportionation is the likely dominating energy and carbon metabolism in incubations with sulfur/lepidocrocite/^13^C-DIC. Sulfur disproportionation becomes an exergonic process when sulfide formed from disproportionation is scavenged by reacting with iron oxides (or Fe^2+^) [72]. This is supported by the strong increase in δ^13^C-TOC in these ^13^C-DIC supplemented incubations, notably in the presence of lepidocrocite, but not sulfur and DIC amended incubations, and a strong increase in RNA-SIP was observed for Loki-2b OTUs (Figs. 2 and S3). In the absence of any genes in MAGs of Loki-2b encoding for known sulfur cycling enzymes (e.g. DSR [73], sox pathway [60], sulfide:quinone reductase [61]), it is most parsimonious to assume that Loki-2b assimilated ^13^C from the primarily labelled bacteria. *Spirochaetaceae* are known degraders of cellulose [57], and thus likely represent the main source of biomass formed in incubations with cellulose and lepidocrocite [57, 60, 61, 71–73]. The indolepyruvate ferredoxin oxidoreductase subunit alpha gene (*iorA)* which had more homologues in Loki-2b than Loki-3 (Fig. 4 and Table S5) was specifically clustered to IOR of MK-D1 in unbinned contigs from ^13^C-labelled DNA (Fig. S11). This result supports the activity of protein degradation by Loki-2b in SIP incubations. According to metagenomic analysis, Loki-2 archaea are equipped to produce H_2_ and short-chain fatty acids during protein fermentation; thus, these substrates are candidate substrates for methanogenesis and sulfate reduction [11] and potentially iron reduction. Beside protein utilization, Loki-2 archaea incorporated ^13^C-labelled inorganic carbon because their RNA had become labelled both, in the presence of unlabelled protein (Fig. 2c); and of unlabelled cellulose (Figs. 2 and 6).

In this study, we have shown that Loki-2 and Loki-3 are mixotrophs but with different patterns of relative abundance across RNA-SIP fractions (for details on mixotrophy criteria, see Supplementary Materials). Loki-2 mixotrophically incorporated organic carbon i.e. protein and inorganic carbon, which is in line with the presence of Loki-2 rRNA in the heavy fractions of incubations amended with ^13^C-protein/DIC and ^13^C-DIC/protein (Fig. 2c). For Loki-3, we did not see an increase of relative abundance in the heaviest fractions but strong increase in the partial labelling fractions in incubations amended with ^13^C-DIC/lignin (Fig. 2a). Overall, we showed how clades of Lokiarchaeota, namely Loki-3 and Loki-2b, differ in their in situ activities by using a systematic series of SIP incubations amended with various carbon substrates including inorganic carbon, aromatic compounds, fermentation products and protein. As revealed by metagenomics, Loki-3 archaea harbour a wide array of metabolic capabilities including inorganic carbon assimilation, lactate utilization and involving in aromatic compound degradation (Fig. 6). On the other hand, Loki-2 appear to thrive uniquely on biomass or protein derived from other microorganisms (Fig. 6). Although both lineages have amino acid degradation in common, SIP revealed that Loki-2b rely more on protein degradation while Loki-3 can alternatively use lactate and participate in aromatic carbon degradation. Equipped with diverse carbon utilization modes, Loki-3 are more widely distributed among marine sediments than Loki-2 (Fig. S12). Thus, this functional divergence in Lokiarchaeotal subgroups may regulate their environmental adaption and global distribution. Our results are the first comprehensive study of the divergent activity and capability of Lokiarchaeotal subgroups, which most likely determine the environmental adaption and distribution of these archaea in marine sediments.

## Supplementary information

Supplementary Materials

Supplementary Tables

## Data Availability

Lokiarchaeotal MAGs and metatranscriptomic data are available at the NCBI database under the project identifiers PRJNA495098, PRJNA360036 and PRJNA505997. Sequencing data of SIP samples have been submitted to Short Reads Archive with accession numbers from SRR8607872 to SRR8607991 and SRR11429462 to SRR11429436. Clone sequences have been deposited at GenBank with accession numbers of MK551261-MK551285.
