# Supplementary material for: Subgroup level differences of physiological activities in marine Lokiarchaeota
Source: ISME J. 2020 Nov 4;15(3):848–61. doi: 10.1038/s41396-020-00818-5 (PMC8027215; doi:10.1038/s41396-020-00818-5)
Supplement: Supplementary file 1 — Supplementary Materials [file 41396_2020_818_MOESM1_ESM.docx]

**Subgroup level differences of physiological activities in marine Lokiarchaeota**

Xiuran Yin^1,2,†^, Mingwei Cai^3,4†^, Yang Liu^3^, Guowei Zhou^1^, Tim Richter-Heitmann^1^, David A. Aromokeye^1,2^, Ajinkya Kulkarni^1,2^, Rolf Nimzyk^1^, Henrik Cullhed^5^, Zhichao Zhou^3,6^, Jie Pan^3^, Yuchun Yang^6^, Ji-Dong Gu^6^, Marcus Elvert^2^, Meng Li^3*^, Michael W. Friedrich^1,2*^

^1^Faculty of Biology/Chemistry, University of Bremen, Bremen, Germany.

^2^MARUM, Center for Marine Environmental Sciences, University of Bremen, Bremen, Germany.

^3^Shenzhen Key Laboratory of Marine Microbiome Engineering, Institute for Advanced Study, Shenzhen University, Shenzhen, China.

^4^Key Laboratory of Optoelectronic Devices and Systems of Ministry of Education and Guangdong Province, College of Optoelectronic Engineering, Shenzhen University, Shenzhen, China.

^5^International Max-Planck Research School for Marine Microbiology, Max Planck Institute for Marine Microbiology, Bremen, Germany

^6^Laboratory of Environmental Microbiology and Toxicology, School of Biological Sciences, The University of Hong Kong, Pokfulam Road, Hong Kong SAR, China.

^*^Correspondence: Michael W. Friedrich, Faculty 02 (Chemistry/Biology) & MARUM, University of Bremen, Leobener Straße 3, D-28359, Bremen, Germany; E-mail: michael.friedrich@uni-bremen.de; Tel: +49-421-218-63060.

Meng Li, Room 360, Administration Building, Institute for Advanced Study, Shenzhen University, Shenzhen, China; E-mail: limeng848@szu.edu.cn; Tel: +86-755-26979250.

Running title: Divergent activities of Lokiarchaeotal subgroups

^†^These authors contributed equally to this work.

Materials and Methods

## Nucleic acids extraction, quantification and DNase treatment

Nucleic acids were extracted according to Lueders et al. [1]. Briefly, 2 mL of wet sediment without supernatant from biological triplicates was used for cell lysis by bead beating, nucleic acid purification by phenol-chloroform-isoamyl alcohol extraction and precipitation with polyethylene glycol. For the RNA extract, DNA was removed by using the RQ1 DNase kit (Promega, Madison, Wisconsin, USA). DNA and RNA were quantified fluorimetrically using Quant-iT PicoGreen and Quant-iT RiboGreen (both Invitrogen, Eugene, Oregon, USA), respectively. In order to obtain enough nucleic acids for SIP, a total volume of 30-50 ml slurries from triplicates were used for both RNA and DNA extraction separately.

**Isopycnic centrifugation and gradient fractionation**

About 0.3–0.7 µg RNA and 4–6.5 µg DNA from SIP incubations were used for DNA- and RNA-SIP, respectively. 0.5 µg of *E.coli* standard (i.e., RNA or DNA) with equal amount of unlabelled and 13C-labelled nucleic acids were used for RNA-SIP and DNA-SIP, respectively. For RNA-SIP, RNA from biological replicates (n=3) was combined and loaded with 240 µL formamide, 6 mL cesium trifluoroacetate solution (CsTFA, GE Healthcare, Buckinghamshire, UK) and gradient buffer solution. The density of the centrifugation medium was adjusted to ~1.80 g mL^-1^ based on refractive index. RNA was density separated by centrifugation at 124 000 *g* at 20 °C for 65 h using an Optima L-90 XP ultracentrifuge with a VTi 65.1 rotor (Beckman Coulter, Brea, California, USA). After ultracentrifugation, 13 fractions were obtained from each sample, and RNA in each fraction was precipitated with 1 volume of isopropanol.

DNA is of lower density than RNA [1], so that the DNA-SIP differs from RNA-SIP. In detail, pooled triplicated DNA extracts in 1.5 ml gradient buffer was mixed with 6.16 ml cesium chloride solution (CsCl, Carl Roth, Karlsruhe, Germany). The density of the mixed medium was adjusted to ~ 1.705 g/ml (refractory index: 1.4020 – 1.4023) with gradient buffer (GB) or CsCl solution. Ultracentrifugation was performed at 192 600 *g* for 40 h at 20 °C with a VTi 65.1 rotor. DNA from 13 fractions was obtained by precipitation with 2 volume of polyethylene glycol (PEG-6000; 30%) and 1 µl of linear polyacrylamide (GenElute; Saint Louis, Missouri, USA). An *E.coli* standard with a mixture of equivalent amount of fully ^13^C-labeled and unlabeled RNA was used to define heavy and light gradient fractions. RNA was quantified and reverse transcription was conducted using the high capacity cDNA reverse transcription kit (Applied Biosystems, Foster City, California, USA).

## Criteria for identifying SIP fractions containing ^13^C-labelled nucleic acid

We have used several criteria to differentiate differentially labelled nucleic acid following recommendations by Lueders [2]:

**I) An increase of relative abundance of OTUs in the heavy fractions between amended incubations and control incubations (inter-gradient comparison).** Here, we compared the SIP results of treatments with both, ^12^C control and ^13^C-DIC controls for Loki-3 OTUs. Considering the fluctuating background of Loki-3 relative abundances in background SIP fractions (i.e., comparsion of ^12^C-DIC and ^13^C-DIC controls), we used a cutoff of > 5% increase of relative abundance in the heavy fractions as the threshold for labelling incorporation. For OTUs with extremely low relative abundances in DIC controls (Loki-2b), the presence of nucleic acid templates in heavy gradient fractions indicated incorporation of ^13^C label.

**II) An increase of relative abundance of OTUs between light and heavy fractions (intra-gradient evaluation).** To further improve resolution of labelling, we sequenced 8 fractions for RNA-SIP (every 2 fractions were pooled, average density reported) along each gradient, representing ultra-light (~1.778-1.789 g/ml), light (~1.790-1.800 g/ml), middle (~1.800-1.809 g/ml) and heavy (~1.810-1.820 g/ml) fractions.

**III**) **Defining “heavy” and “light” gradient fractions” by standardization with RNA and DNA of fully labelled and unlabeled *E. coli* standards (Fig. S2**). From the RNA-SIP profiles of the *E. coli* standard, the unlabelled peak had a density of ~1.790 g/ml and fully ^13^C-labelled peak at ~1.823 g/ml. For DNA-SIP, unlabeled DNA peaked at 1.696 g/ml and the fully labelled DNA peaked at 1.712 g/ml. Our density values are consistent with those reported in the literature [1] but certainly depend on the centrifugation system.

The background of templates in all SIP gradient separations, which can be critical in DNA-SIP studies [2], was evaluated as follows. For RNA-SIP, the mol% G+C effect is not critical as the range of G+C contents in rRNA is limited to 50-60% [2]. For DNA-SIP, controls with unlabeled substrate (“^12^C”) were performed and showed a maximum of 1.707 g/ml for the distribution of natural Lokiarchaeota DNA (Fig.2), which corroborates with the low G+C DNA content (30 mol%) observed in Lokiarchaeota MAGs in our study (Table S2) and in other studies [3]. Thus, an interference of Lokiarchaeota GC smear into heavy gradient fractions (>1.707 g/ml) can be excluded.

In addition, a “subtraction method modified from Zumsteg et al. [4] was used to infer the relative increase of target OTUs in “heavy” fractions compared to the representation in “light” fractions of the same gradient (intra-gradient comparison).

Clone library construction

A clone library of archaeal 16S rRNA gene fragments (>800 bp) was constructed for improving the resolution of our phylogenetic analysis. The DNA-SIP samples from heavy fractions of the incubations amended with sulfur and lepidocrocite (density = 1.715 g/ml), lignin and lepidocrocite (1.714 g/ml) as well as humic acid and lepidocrocite (1.714 g/ml) were used for cloning. According to the criteria for identifying SIP fractions containing 13C-labelled nucleic acid, these fractions used for cloning belonged to the heavy fractions. PCR was performed with the primer set of Arc8F (5’-TCCGGTTGATCCTGCC-3’)/Arc912R (5’-GTGCTCCCCCGCCAATTCCTTTA-3’) [5, 6] using ALLin RPH polymerase Kit (highQu, Kraichtal, Germany). Thermocycling was as follows: 95 °C for 3 min; 40 cycles at 95 °C for 30 sec, 55 °C for 45 sec and 72 °C for 45 sec; 72 °C for 10 min. Purified PCR products were ligated into the pGEM-T vector (Promega, Mannheim, Germany) and transformed into Escherichia coli JM109 competent cells (Promega, Mannheim, Germany) according to the manufacturer. White colonies were randomly picked and cell material was directly subjected to colony PCR with the following cycling parameters: 95 °C for 5 min; 28 cycles at 95 °C for 30 sec, 55 °C for 45 sec, and 72 °C for 1 min; 72 °C for 5 min. Amplicons of 96 clones were submitted to LGC Genomics (Berlin, Germany) for Sanger sequencing. Clone sequences have been deposited at GenBank with accession numbers of MK551261-MK551285.

## Mangrove sediment collection, metagenomics and metatranscriptomics

Samples were collected from the coastal sediment of China (Table S2). They were sampled using custom cores, sealed in plastic bags in duplicates, stored in a sampling box with ice bags and transported to the lab within 4 hours. The physiochemical parameters of the samples were determined as previously described [7]. Samples for RNA extraction were preserved in RNALater (Ambion, Life Technologies). For each sample, 10 g sediment each was used for DNA and RNA isolation with the PowerSoil DNA Isolation Kit (MO BIO) and RNA Powersoil™ Total RNA Isolation Kit (QIAGEN), respectively. The rRNA genes were removed from the total RNA using Ribo-Zero rRNA removal kit (Illumina, Inc., San Diego, CA, USA) and the remaining mRNA was reverse-transcribed. DNA and cDNA were sequenced using an Illumina HiSeq sequencer (Illumina) with 150-bp paired-end reads at BerryGenomics (Beijing, China). Metatranscriptomic reads were quality-trimmed using Sickle (version 1.33) with the quality score ≥25, and the potential rRNA reads were removed using SortMeRNA (version 2.0) [8] against both the SILVA 132 database and the default databases (E-value cutoff ≤1e-5).

Metabolic pathways were reconstructed based on the predicted annotations and the reference pathways depicted in KEGG and MetaCyc [9]. Metatranscriptome data from mangrove and mudflat sediments of Shenzhen Bay were analyzed to clarify the transcriptomic activity of Lokiarchaeota. The abundance of transcripts for each gene was determined by mapping all non-rRNA transcripts to the predicted genes using BWA with default setting [10, 11]. Normalized expression was expressed in transcript per million units (TPM), followed by normalization by genome number (Table S6).

## Phylogenetic analysis of functional genes

Amino acid sequences including lactate dehydrogenase, lactate utilization enzyme A, lactate utilization enzyme B and multiple ligases (long-chain-fatty-acid-CoA, 4-coumarate-CoA and phenylacetate-CoA ligase) were used for orthology analysis. Reference sequences were retrieved from NCBI non-redundant protein database using Lokiarchaeota sequences obtained from DNA-SIP and sediment samples of South China Sea. The combined sequences of each protein were filtered and clustered using cd-hit (Version 4.6.8) [12] with cut-off of 65 to 70% separately, which was followed by MUSCLE (Version 3.8.31) alignment with default parameters and trim by BMGE with flags “-t AA -m BLOSUM30” [13]. Un-rooted phylogenetic trees for protein sequence were built with 1000 times ultrafast bootstrapping using IQ-TREE with the best-fit models based on estimation by ModelFinder [14].

## Mixotrophy criteria

Different modes of mixotrophy exist, e.g., facultative mixotrophy and obligate mixotrophy. If we assume that Lokiarcheota are obligate mixotrophs, DIC and organic carbon are used simultaneously. In labelling experiments, this should result in maximum labelling therefore only, when both substrates are provided labelled. This was tested in incubations with ^13^C-DIC and ^13^C-labelled protein, and variations thereof. Strongest labelling of Loki-2b was observed when ^13^C-DIC and ^13^C-labelled protein were simultaneously present in incubations.

Facultative mixotrophy of Lokiarchaea: the optional use of DIC or organic carbon, can be observed when either one, ^13^C-DIC, or ^13^C-organic carbon, is provided labelled. This scenario was tested in the majority of incubations, e.g. ^13^C-DIC (plus different types of carbon compounds or electron donors, e.g. sulfur). Especially for RNA-SIP incubations amended with ^13^C-DIC/lignin, we saw a partial labelling in RNA of Loki-3 (Fig.2a). Full labelling of Loki-3 RNA would have required that organic carbon amended (e.g. lignin, or humics) was also fully ^13^C-labelled, which was not the case. The observed partial labelling of Loki-3 RNA is parsimoniously explained by a maximum of 40% ^13^C labelling from ^13^C-DIC based on the nucleic acid pathway in Lokiarchaeota (Fig. 5).


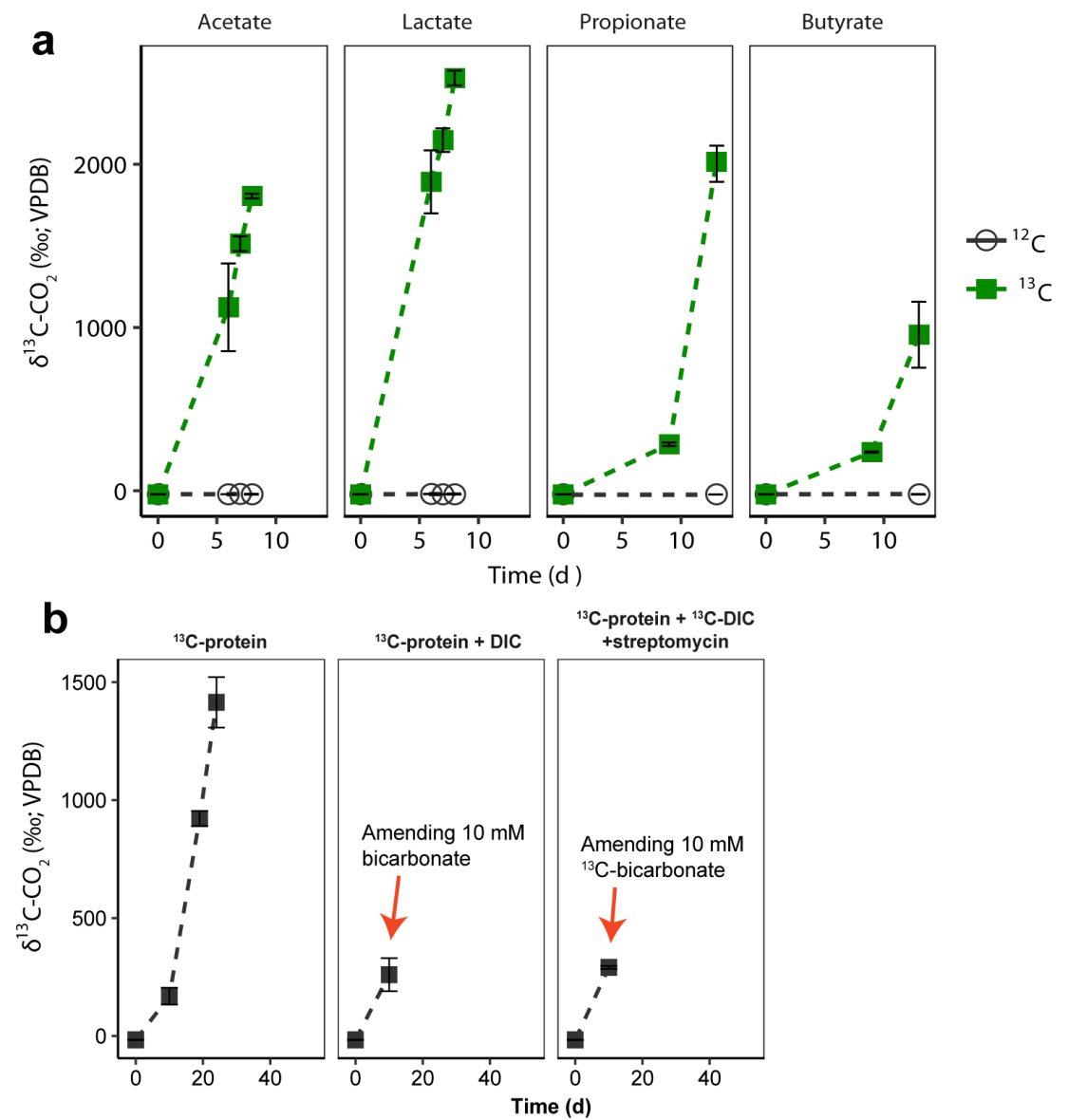


**Fig. S1** Development of δ^13^C-CO_2_ in RNA-SIP incubations amended with ^13^C-labelled and unlabelled fermentation intermediates **(a)** and protein **(b)**. Incubations were stopped after 24 day.


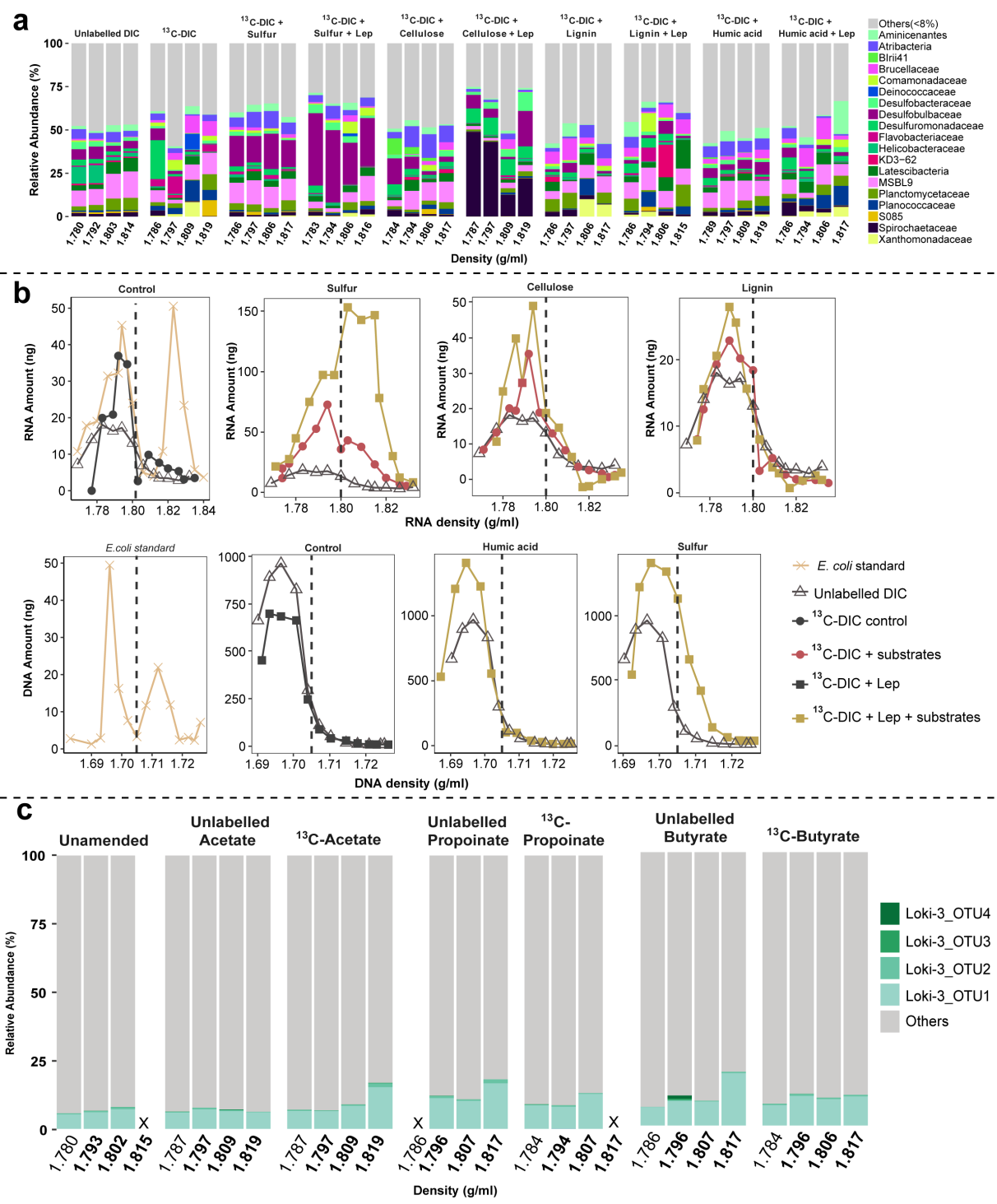


**Fig. S2** (a) Total sum scaling of abundances of bacterial 16S rRNA gene sequences from selected “light” and “heavy” gradient fractions of RNA-SIP samples. Before Illumina sequencing, two fractions (fraction 4 and 5, 6 and 7, 8 and 9, 10 and 11, respectively) were combined together as one sample for library preparation. Density indicates the average density of combined fractions for RNA-SIP samples. Relative abundances are shown at the family level as taxonomic threshold. (b) RNA-SIP profiles and DNA-SIP profiles. RNA-SIP profile of *E.coli* standard was performed by ultra-centrifuging the equal amount of mixed unlabelled and 13C-labelled RNA of *E.coli*. (c) Relative abundance of Lokiarchaeota in total Archaea reads from RNA-SIP samples. X denotes that cDNA synthesis failed because of the low amount of RNA in these fractions. DIC: dissolved inorganic carbon (i.e. bicarbonate); S: sulfur; Lep: lepidocrocite. Dashed lines indicate starting density before ultracentrifugation.


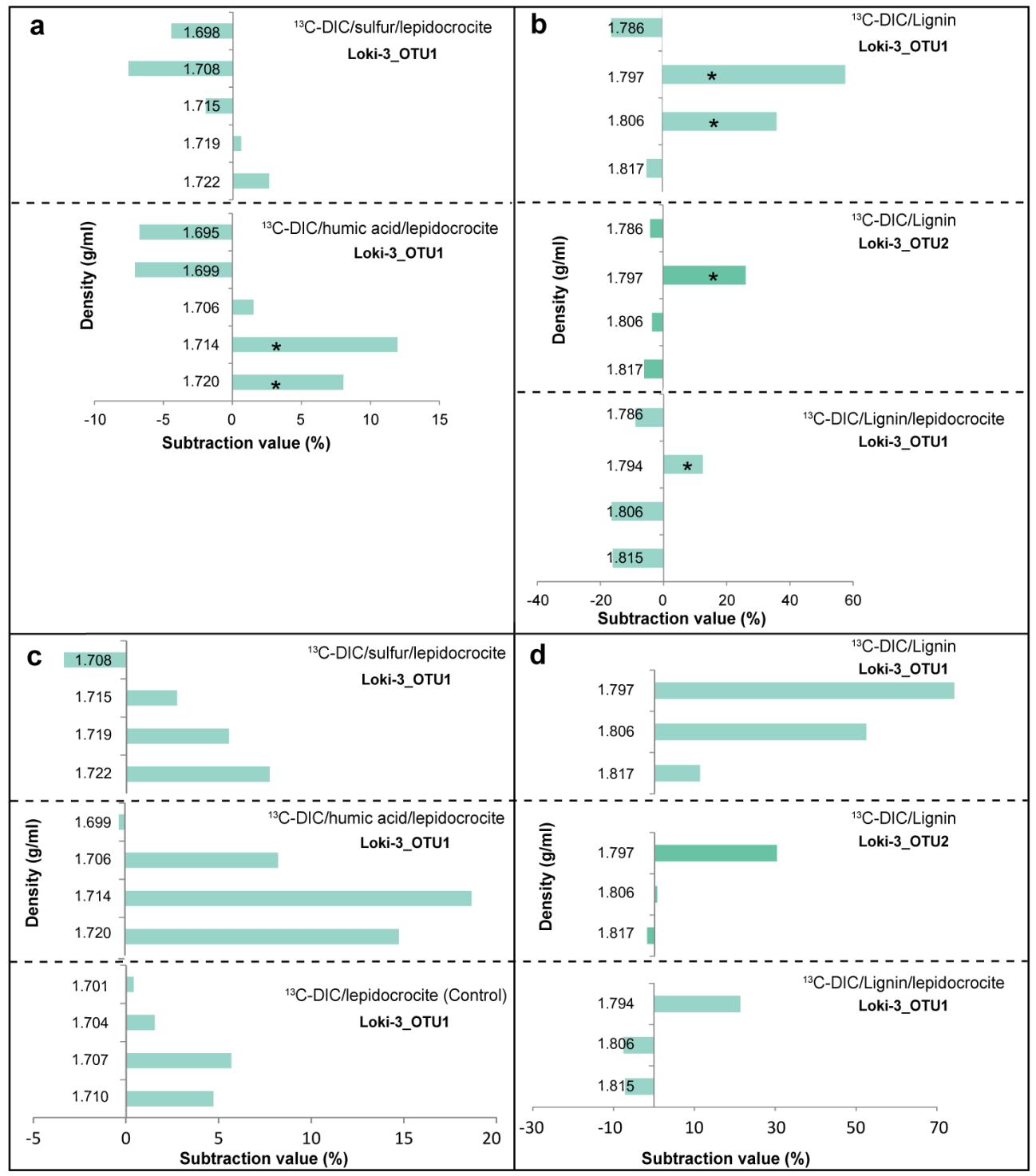


Fig. S3. Inter-gradient subtraction values of target populations using the relative abundances of ^13^C-treatment incubations compared to the maximum relative abundance of DIC control incubations (both unlabelled DIC and ^13^C-DIC) for DNA-SIP (a) and RNA-SIP (b). Intra-gradient subtraction values of target populations using fraction with lowest density compared to the other fractions within one gradient for DNA-SIP (c) and RNA-SIP (d). A minimum of 5% increase for subtraction values was regarded as community shift. * indicates inter-gradient increase.


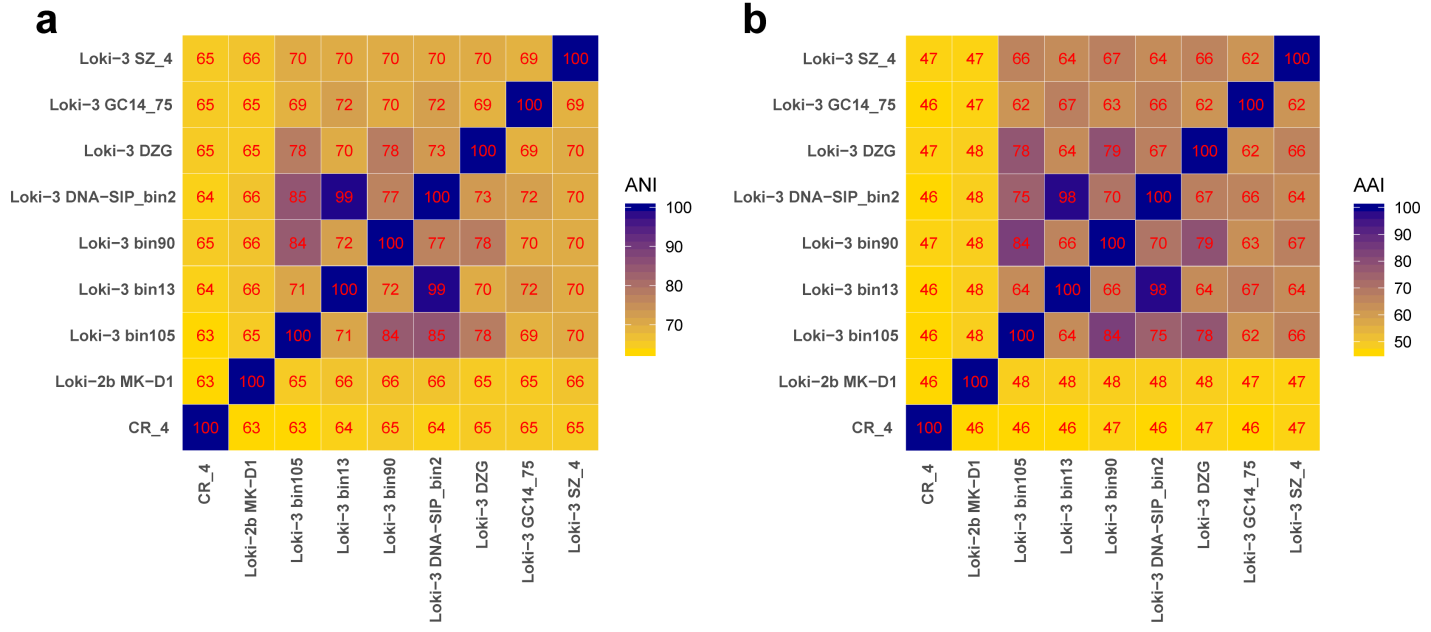


Fig. S4 (a) Average nucleotide identity (ANI) and (b) amino acid identity (AAI) of different Lokiarchaeotal subgroups. ANI and AAI was calculated using OrthoANI [12] and CompareM (<https://github.com/dparks1134/CompareM>), respectively.


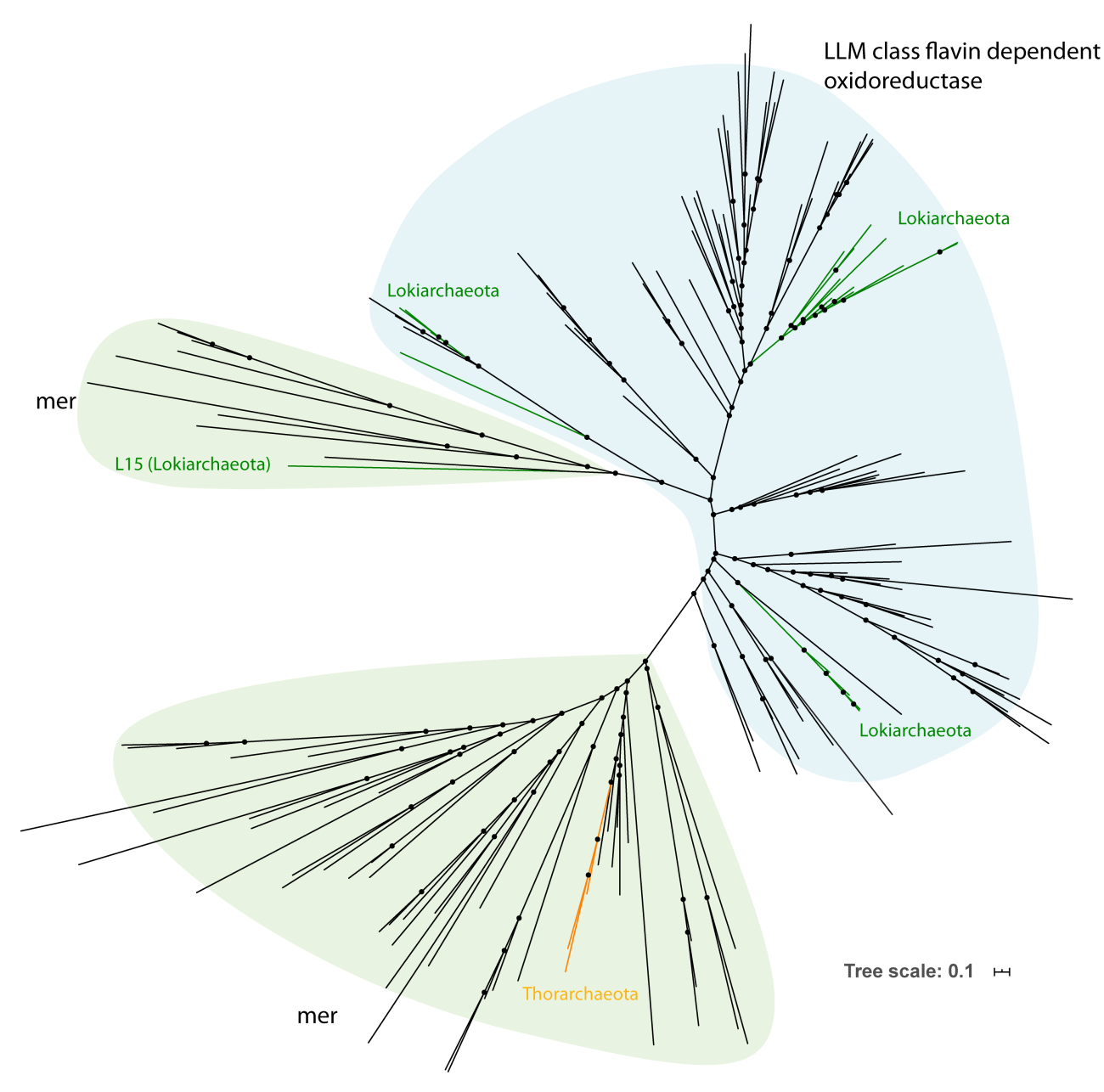


**Fig. S5** Maximum likelihood phylogenetic analyses of protein (COG2141) closed to 5,10-methylenetetrahydromethanopterin reductase (mer). The unrooted phylogeny inferred from an alignment consisting of 146 amino acid positions with LG+R6 model. LLM: luciferase-like monooxygenase. Light blue area: LLM class flavin dependent oxidoreductase. Green area: mer.

**
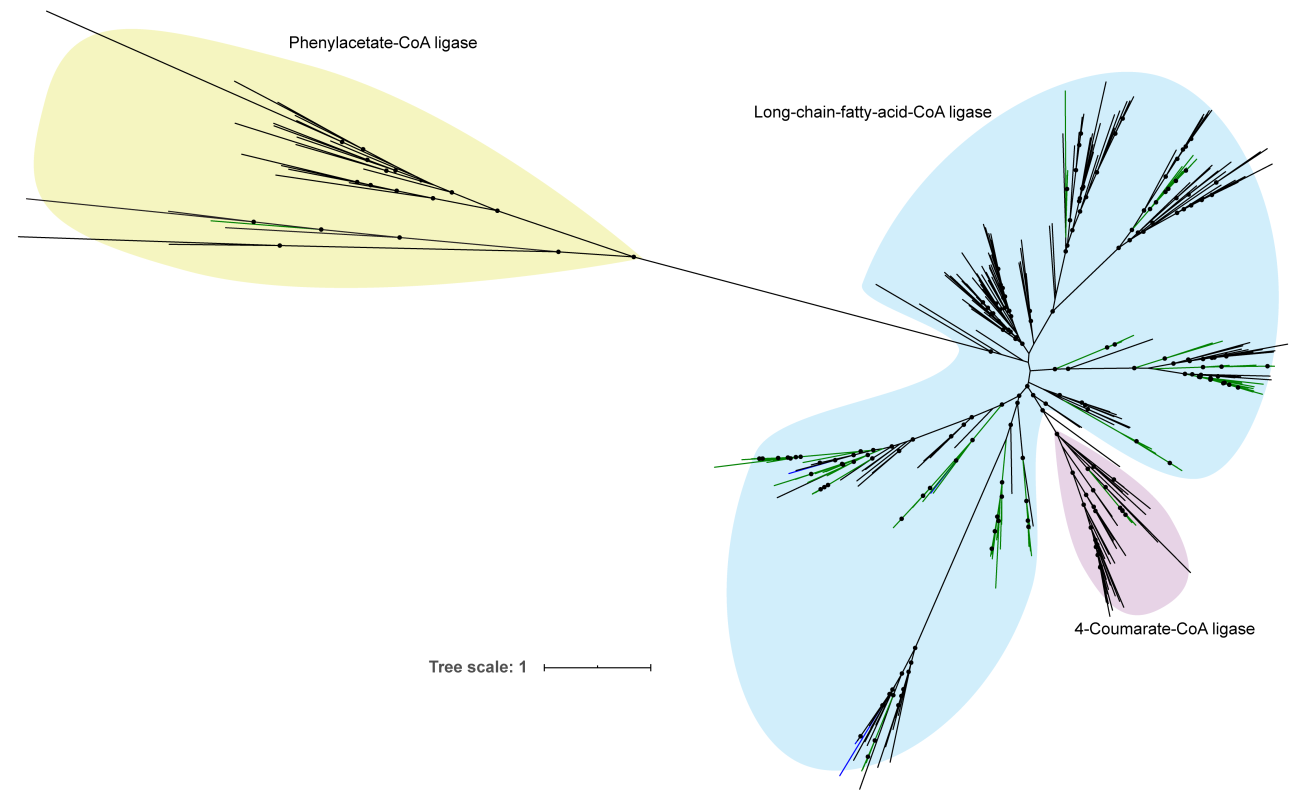
**

**Fig. S6** Maximum likelihood phylogenetic analyses of multiple coenzyme A ligases (long-chain-fatty-acid-CoA, 4-coumarate-CoA and phenylacetate-CoA ligase). The unrooted phylogeny inferred from an alignment consisting of 249 amino acid positions with LG+R7 model. Yellow area: phenylacetate-CoA ligase; light blue area; long-chain-fatty-acid-CoA ligase; pink area: 4-coumarate-CoA ligase. Blue branch: MK-D1 from enrichment [13]; Green branch: Loki-3 from our study.

##
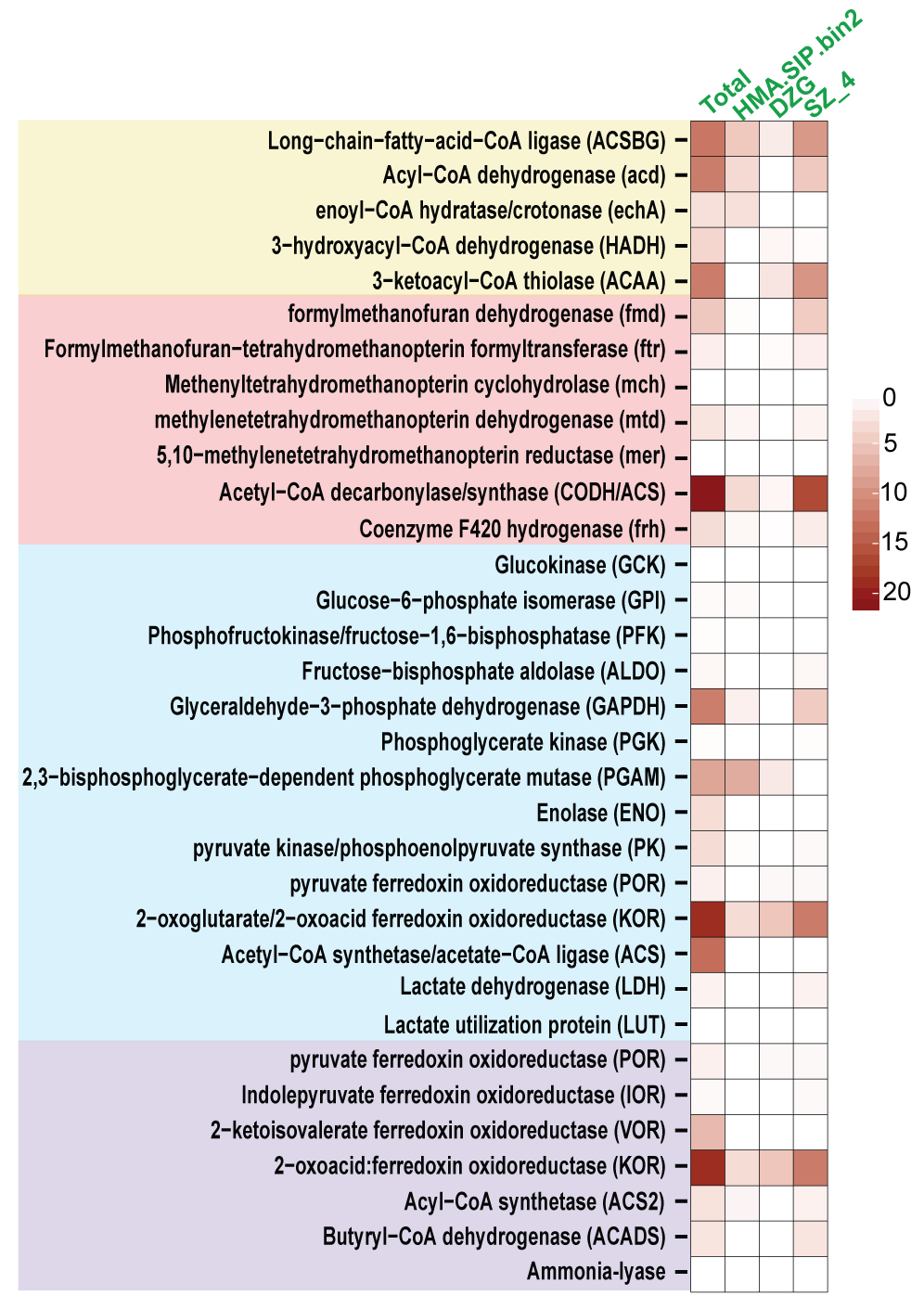


**Fig. S7** Metatranscriptomic analysis of original mangrove sediment from South China. Transcripts per kilobase million (TMP) indicates the summed value of the same transcripts mapped by different Lokiarchaeotal MAGs. Table: summed TMP based on analysis using the three MAGs.

**
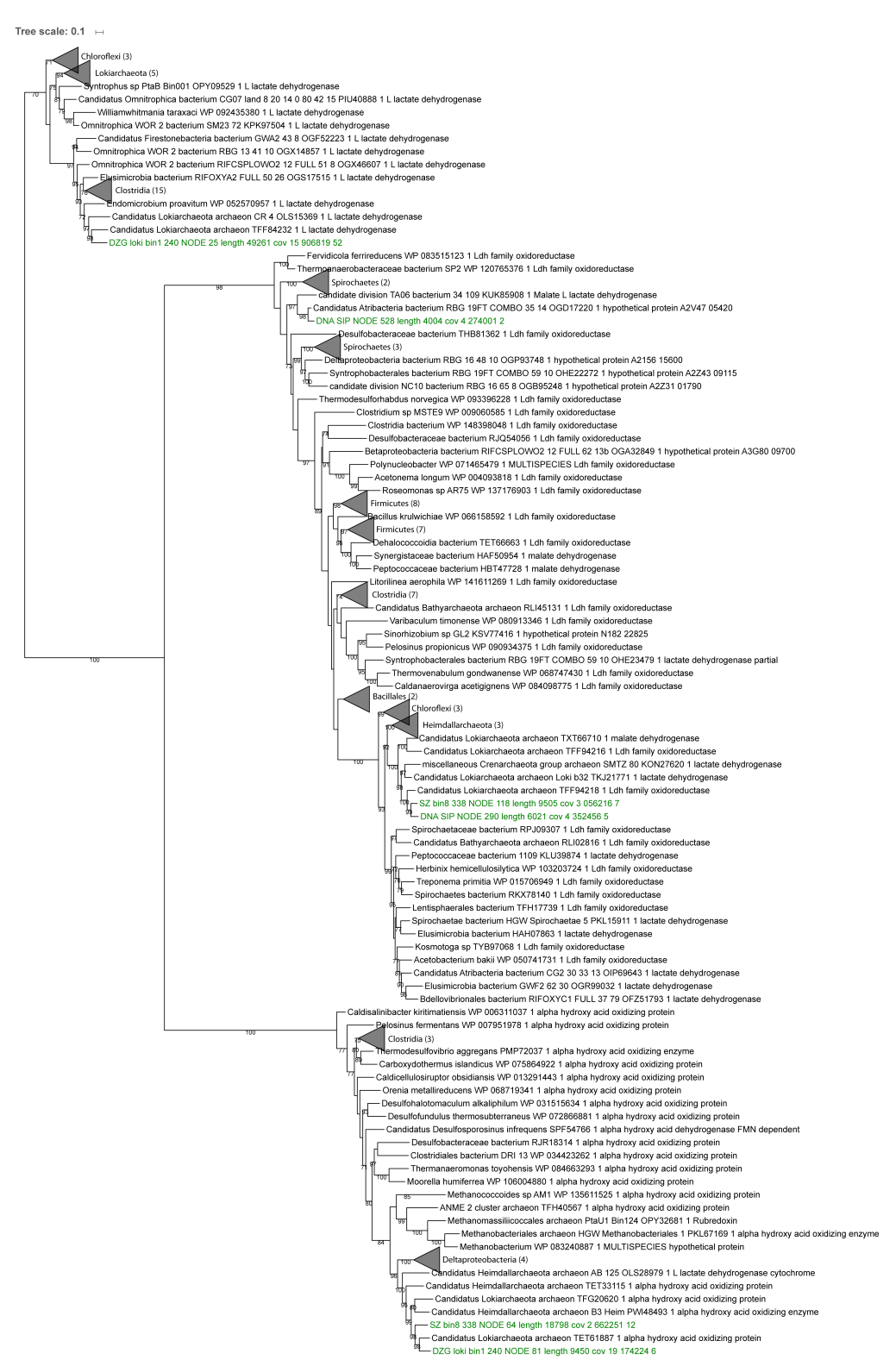
**

**Fig. S8** Maximum likelihood phylogenetic analyses of lactate dehydrogenase. The unrooted phylogeny inferred from an alignment consisting of 219 amino acid positions with LG+R4 model.


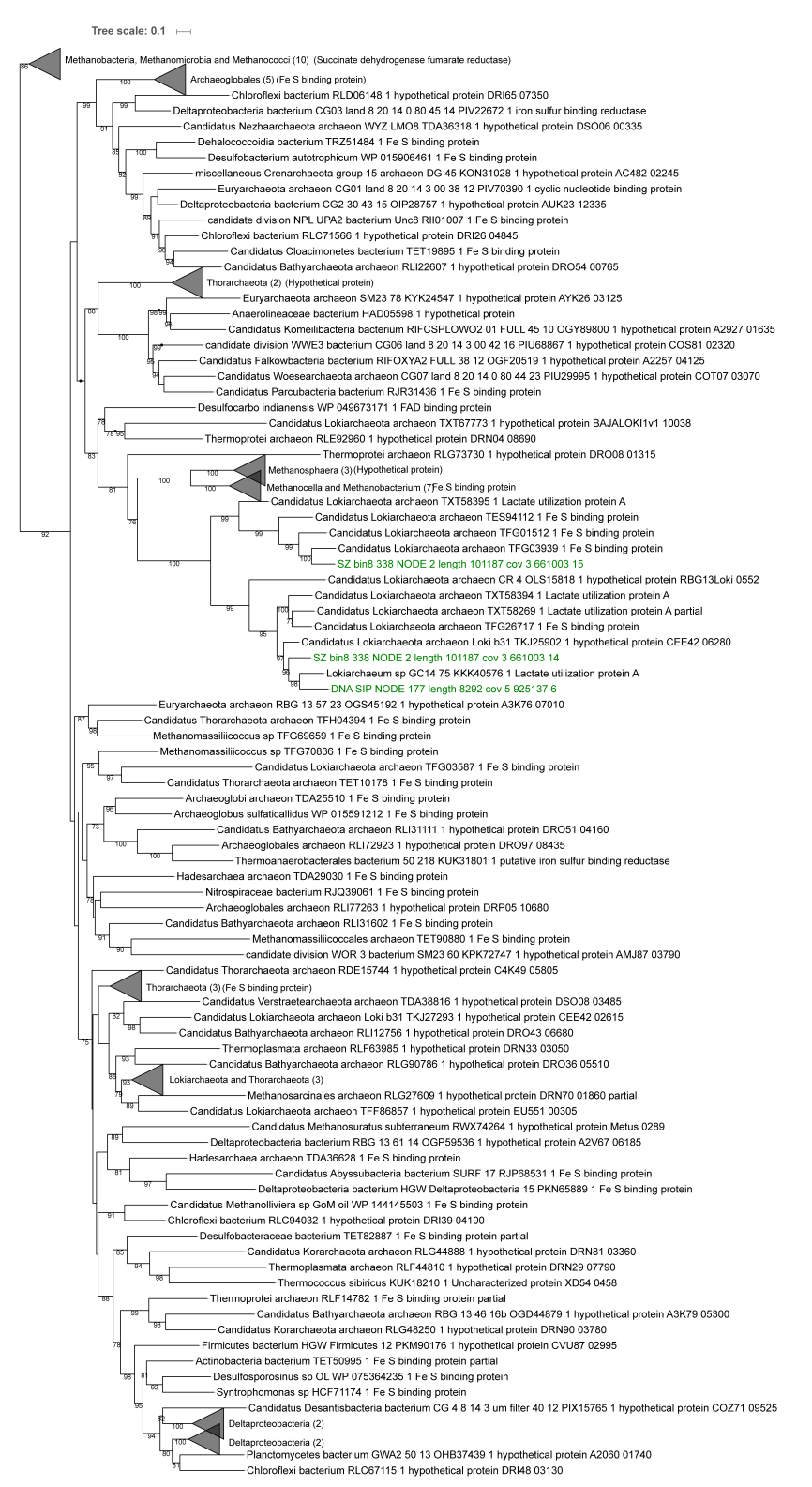


**Fig. S9** Maximum likelihood phylogenetic analyses of lactate utilization protein A. The unrooted phylogeny inferred from an alignment consisting of 152 amino acid positions with LG+R6 model.


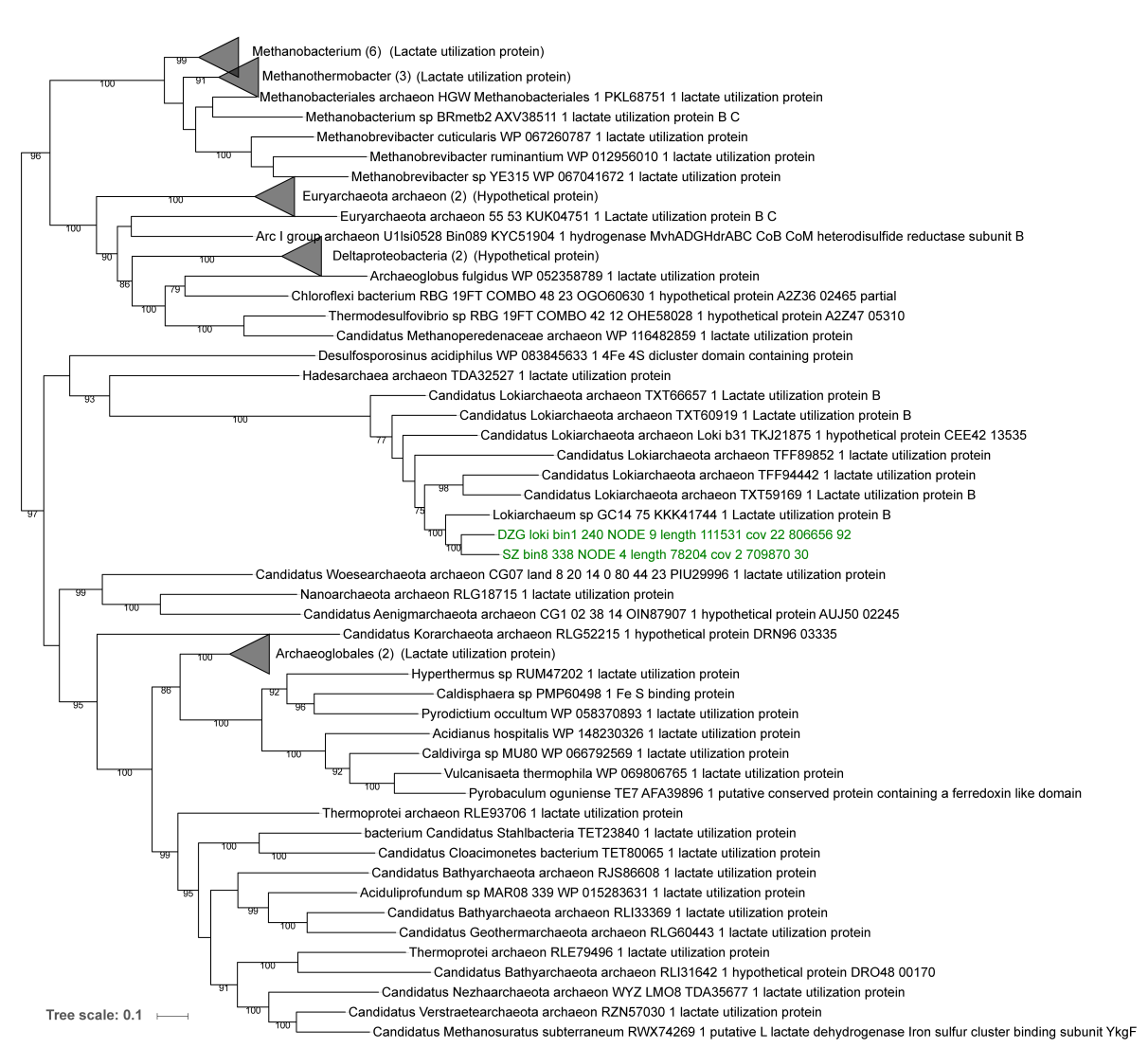


**Fig. S10** Maximum likelihood phylogenetic analyses of lactate utilization protein B. The unrooted phylogeny inferred from an alignment consisting of 327 amino acid positions with LG+R5 model.

**
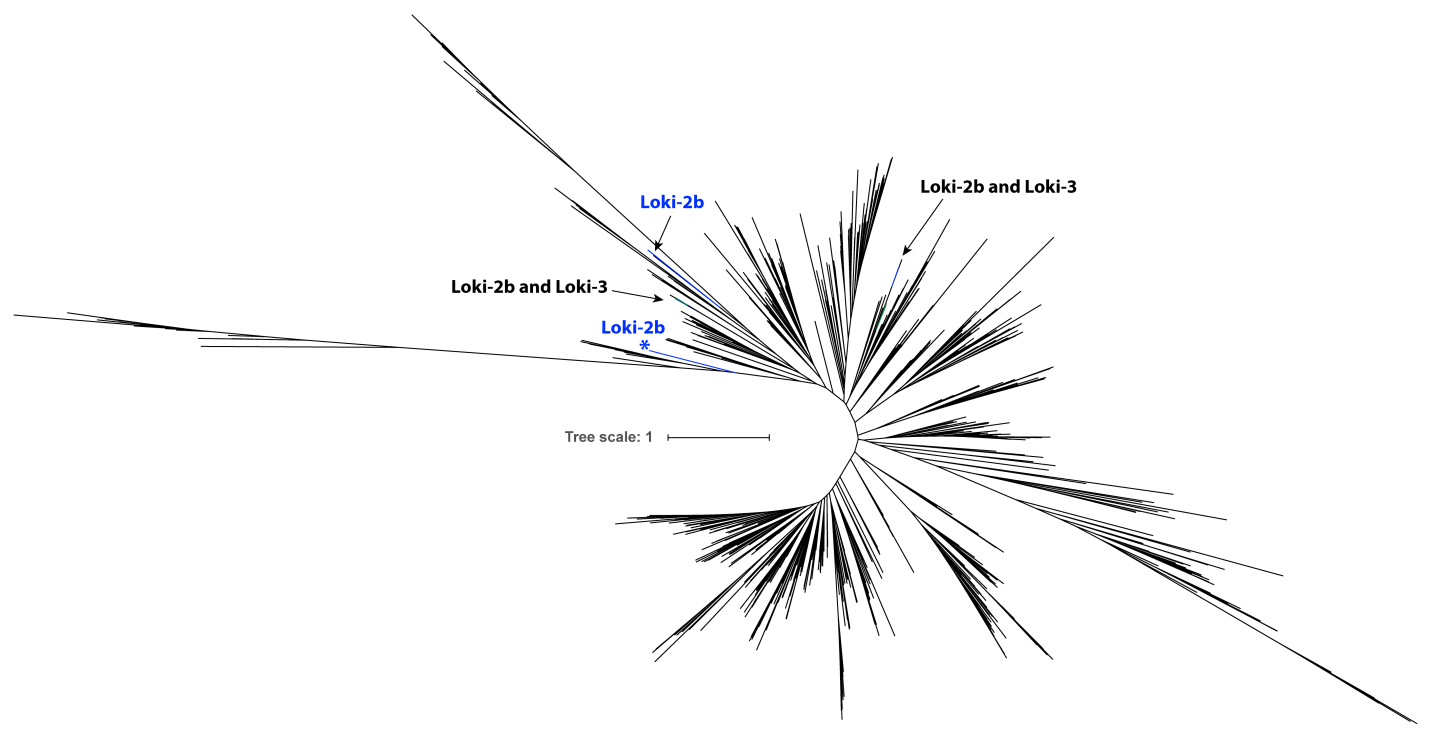
**

**Fig. S11** Maximum likelihood phylogenetic analyses of indolepyruvate ferredoxin oxidoreductase subunit alpha (*iorA*) gene. The unrooted phylogeny inferred from an alignment consisting of 258 amino acid positions with LG+F+R9 model. * indicates *IorA* gene of MK-D1 and *IorA* gene sequence obtained from unbinned contigs from ^13^C-DNA from amended with sulfur, lepidocrocite and ^13^C-labelled bicarbonate.


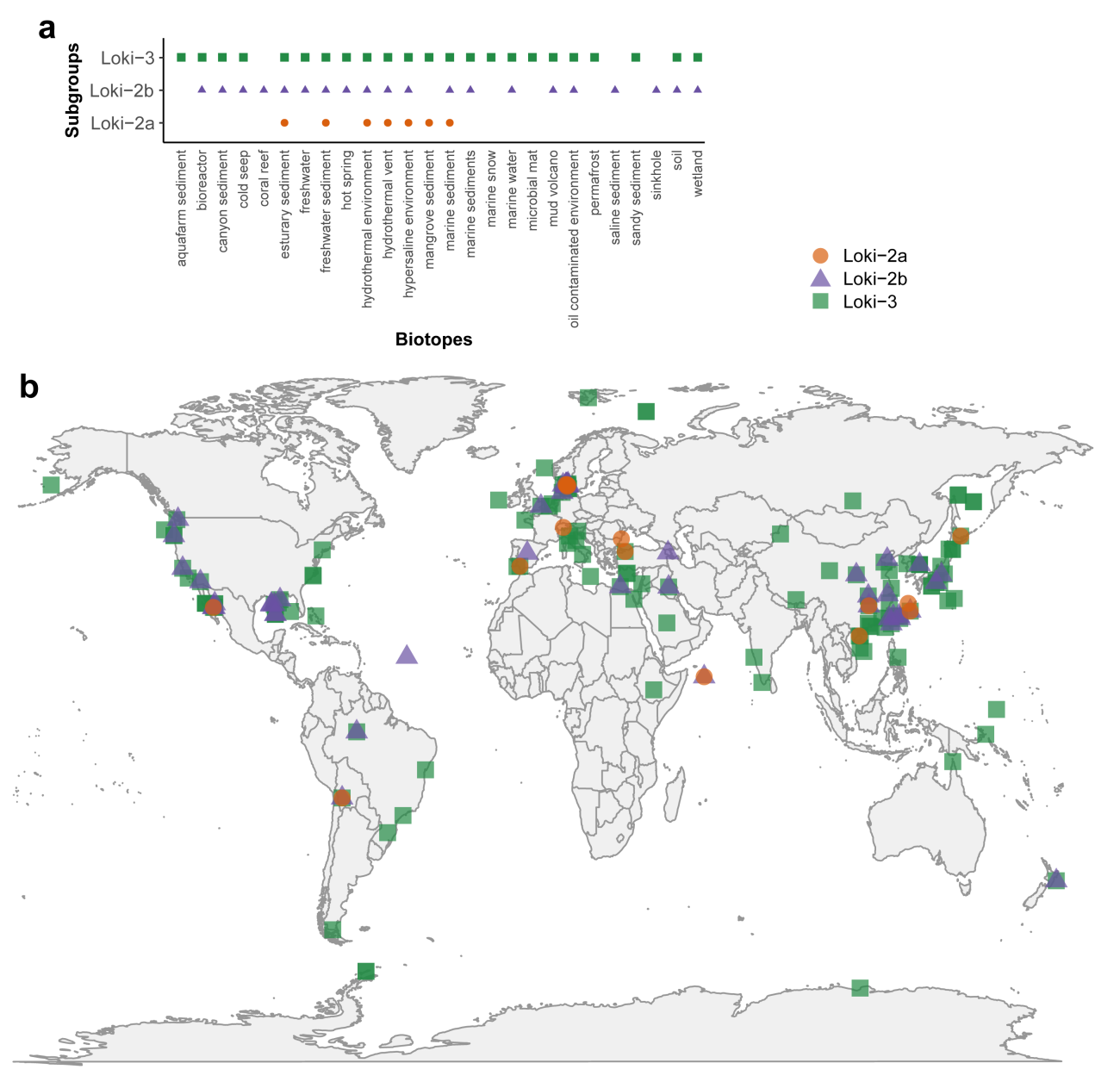


**Fig. S12** Biotopes (a) and global distribution (b) of Loki-2a, Loki-2b and Loki-3.

## References

1. Lueders T, Manefield M, Friedrich MW. Enhanced sensitivity of DNA- and rRNA-based stable isotope probing by fractionation and quantitative analysis of isopycnic centrifugation gradients. Environ Microbiol. 2003;6:73-8.

2. Lueders T. DNA- and RNA-Based stable isotope probing of hydrocarbon degraders. Hydrocarbon and Lipid Microbiology Protocols. Springer Protocols Handbooks 2015. p. 181-97.

3. Caceres EF, Lewis WH, Homa F, Martin T, Schramm A, Kjeldsen KU, et al. Near-complete Lokiarchaeota genomes from complex environmental samples using long and short read metagenomic analyses. BioRxiv preprint. 2019;doi.org/10.1101/2019.12.17.879148

4. Zumsteg A, Schmutz S, Frey B. Identification of biomass utilizing bacteria in a carbon-depleted glacier forefield soil by the use of ^13^C DNA stable isotope probing. Environ Microbiol Rep. 2013;5:424-37.

5. Lloyd KG, Lapham L, Teske A. An anaerobic methane-oxidizing community of ANME-1b archaea in hypersaline Gulf of Mexico sediments. Appl Environ Microbiol. 2006;72:7218-30.

6. Lueders T, Friedrich MW. Effects of amendment with ferrihydrite and gypsum on the structure and activity of methanogenic populations in rice field soil. Appl Environ Microbiol. 2002;68:2484-94.

7. Zhou Z, Meng H, Liu Y, Gu JD, Li M. Stratified Bacterial and Archaeal Community in Mangrove and Intertidal Wetland Mudflats Revealed by High Throughput 16S rRNA Gene Sequencing. Front Microbiol. 2017;8:2148.

8. Kopylova E, Noé L, Touzet H. SortMeRNA: fast and accurate filtering of ribosomal RNAs in metatranscriptomic data. Bioinformatics. 2012;28:3211-7.

9. Caspi R, Billington R, Ferrer L, Foerster H, Fulcher CA, Keseler IM, et al. The MetaCyc database of metabolic pathways and enzymes and the BioCyc collection of pathway/genome databases. Nucleic Acids Research. 2015;44:D471-D80.

10. Li H, Durbin R. Fast and accurate long-read alignment with Burrows-Wheeler transform. Bioinformatics. 2010;26:589-95.

11. Li M, Baker BJ, Anantharaman K, Jain S, Breier JA, Dick GJ. Genomic and transcriptomic evidence for scavenging of diverse organic compounds by widespread deep-sea archaea. Nat Commun. 2015;6:8933.

12. Fu L, Niu B, Zhu Z, Wu S, Li W. CD-HIT: accelerated for clustering the next-generation sequencing data. Bioinformatics. 2012;28:3150-2.

13. Criscuolo A, Gribaldo S. BMGE (Block Mapping and Gathering with Entropy): a new software for selection of phylogenetic informative regions from multiple sequence alignments. BMC Evolutionary Biology. 2010;10:210.

14. Nguyen LT, Schmidt HA, von Haeseler A, Minh BQ. IQ-TREE: a fast and effective stochastic algorithm for estimating maximum-likelihood phylogenies. Mol Biol Evol. 2015;32:268-74.
